# Recovery of an Antiviral Antibody Response following Attrition Caused by Unrelated Infection

**DOI:** 10.1371/journal.ppat.1003843

**Published:** 2014-01-02

**Authors:** Dorothy H. L. Ng, John J. Skehel, George Kassiotis, Jean Langhorne

**Affiliations:** 1 Division of Immunoregulation, MRC National Institute for Medical Research, London, United Kingdom; 2 Division of Parasitology, MRC National Institute for Medical Research, London, United Kingdom; 3 Division of Virology, MRC National Institute for Medical Research, London, United Kingdom; London School of Hygiene and Tropical Medicine, United Kingdom

## Abstract

The homeostatic mechanisms that regulate the maintenance of immunological memory to the multiple pathogen encounters over time are unknown. We found that a single malaria episode caused significant dysregulation of pre-established Influenza A virus-specific long-lived plasma cells (LLPCs) resulting in the loss of Influenza A virus-specific Abs and increased susceptibility to Influenza A virus re-infection. This loss of LLPCs involved an FcγRIIB-dependent mechanism, leading to their apoptosis. However, given enough time following malaria, the LLPC pool and humoral immunity to Influenza A virus were eventually restored. Supporting a role for continuous conversion of Influenza A virus-specific B into LLPCs in the restoration of Influenza A virus immunity, B cell depletion experiments also demonstrated a similar requirement for the long-term maintenance of serum Influenza A virus-specific Abs in an intact LLPC compartment. These findings show that, in addition to their established role in the anamnestic response to reinfection, the B cell pool continues to be a major contributor to the maintenance of long-term humoral immunity following primary Influenza A virus infection, and to the recovery from attrition following heterologous infection. These data have implications for understanding the longevity of protective efficacy of vaccinations in countries where continuous infections are endemic.

## Author Summary

Antibody responses to infectious pathogens are critical in host survival, recovery and protection from reinfection; they also correlate with the success of vaccination. It is currently thought that antibody serum titers are maintained at protective levels over long periods of time by specialized long-lived antibody-secreting plasma cells residing in the bone marrow. Indeed, antibodies against the original virus can still be found in survivors of the 1918 Spanish Flu, more than 90 years ago. However, it is also becoming clear that subsequent infection with heterologous pathogens may cause attrition of previously established immunological memory, in order to accommodate new lymphocyte specificities in the finite space of the host. This phenomenon is seemingly at odds with long-term maintenance of immunological memory. We also show that a single episode of malaria, caused by infection by *Plasmodium chabaudi*, leads to the loss of preexisting plasma cells, serum antibodies and protective immunity against Influenza A virus. However, Influenza A virus-specific immunity does eventually recover in these animals with the replenishment of plasma cells by B cells over the course of several weeks. Thus, the reported mechanism reconciles attrition of immunological memory by heterologous infection and long-term stability, and places B cells, instead of their descendant plasma cells, at the center of humoral memory.

## Introduction

Infection or vaccination usually induces high levels of antigen-specific antibodies (Abs) in the systemic circulation and mucosal surfaces. These Abs can be maintained for long periods of time in the absence of re-infection, despite the relatively short half-life of serum immunoglobulins, which is measured in weeks [1]. For example, virus-neutralizing Abs have been detected in humans over 90 years after Influenza A virus infection [2] and in mice over 250 days after lymphocytic choriomeningitis virus (LCMV) infection [3]. The establishment of these long-term Ab responses relies on the maintenance of antigen-specific memory B cells (MBCs) and long-lived plasma cells (LLPCs). MBCs and LLPCs occupy distinct anatomical locations in the spleen and bone marrow, respectively, which are thought to be of finite size and under homeostatic control [4]. One consequence of such regulation is that new antigenic challenges, particularly with complex pathogens that generate large populations of MBCs and LLPCs, would affect the maintenance of Ab responses to previously encountered antigens.

Infection with the malaria parasite, *Plasmodium*, has long been known to induce a strong B cell response, giving rise to large numbers of LLPCs [5], hypergammaglobulinemia in humans [6] and in experimental models [7], [8], and perturbations of splenic and bone marrow microarchitecture [7], [9]. Ab responses, MBCs or LLPCs specific for antigens administered prior to or during an experimental blood-stage malaria infection can be delayed, and/or reduced in magnitude and avidity [8], [10], [11]. Similar observations were made after *Trypanosoma brucei* infection of mice, which caused a reduction in pre-established MBCs and LLPCs and an increase in susceptibility to heterologous infection [12].

The mechanisms by which subsequent infections may cause the attrition of pre-existing heterologous MBCs and LLPCs are not entirely understood. Apoptosis of pre-existing parasite-specific and unrelated MBCs and LLPCs has been described in non-lethal rodent *Plasmodium* strain *P. yoelii* [10]. Immune complexes cross-linking of the inhibitory receptor FcγRIIB on the surface of LLPCs have been shown to induce apoptosis of LLPCs in the bone marrow that were induced by protein immunization [13]. However, it is currently unclear whether or not similar mechanisms underlie loss of pre-established humoral immunity following protein immunization or parasitic infection.

Loss of pre-existing heterologous humoral immunity following parasitic infections has been documented extensively [10]–[12], [14] and is seemingly at odds with long-term maintenance of antiviral Abs [15]. Therefore we examined more closely both the kinetics and potential mechanisms of humoral memory attrition. Here, we investigated whether the blood stages of the malaria parasite would affect pre-established humoral immunity to Influenza A virus. We established a mouse model of sequential infection with Influenza A/Puerto Rico/8/34 (PR8) and the rodent malaria parasite *Plasmodium chabaudi chabaudi* (AS). We found that sequential infection of PR8-immune mice with *P. chabaudi* resulted in the loss of pre-established serum PR8-specific Abs and LLPCs in the bone marrow, and this rendered mice more susceptible to PR8 challenge. Moreover, during *P. chabaudi* infection, LLPCs underwent apoptosis in the bone marrow, through an FcγRIIB-dependent mechanism. However, the loss of pre-established humoral immunity was temporary, as antiviral serum Abs and LLPC numbers did eventually return to levels observed before the *P. chabaudi* infection. Importantly, B cells were essential for the maintenance of long-lived serum Ab titers to PR8, as B cell depletion in PR8-immune mice resulted in the eventual loss, without recovery, of LLPCs and antiviral serum Abs. These results confirm the detrimental effect of parasitic infection on the LLPC pool and serum titers of antiviral antibodies, which is eventually restored by further LLPC generation, thus reconciling humoral memory attrition by subsequent infection and long-term stability.

## Results

### Loss of pre-established humoral immunity after infection with *P. chabaudi*

**Figure 1:**
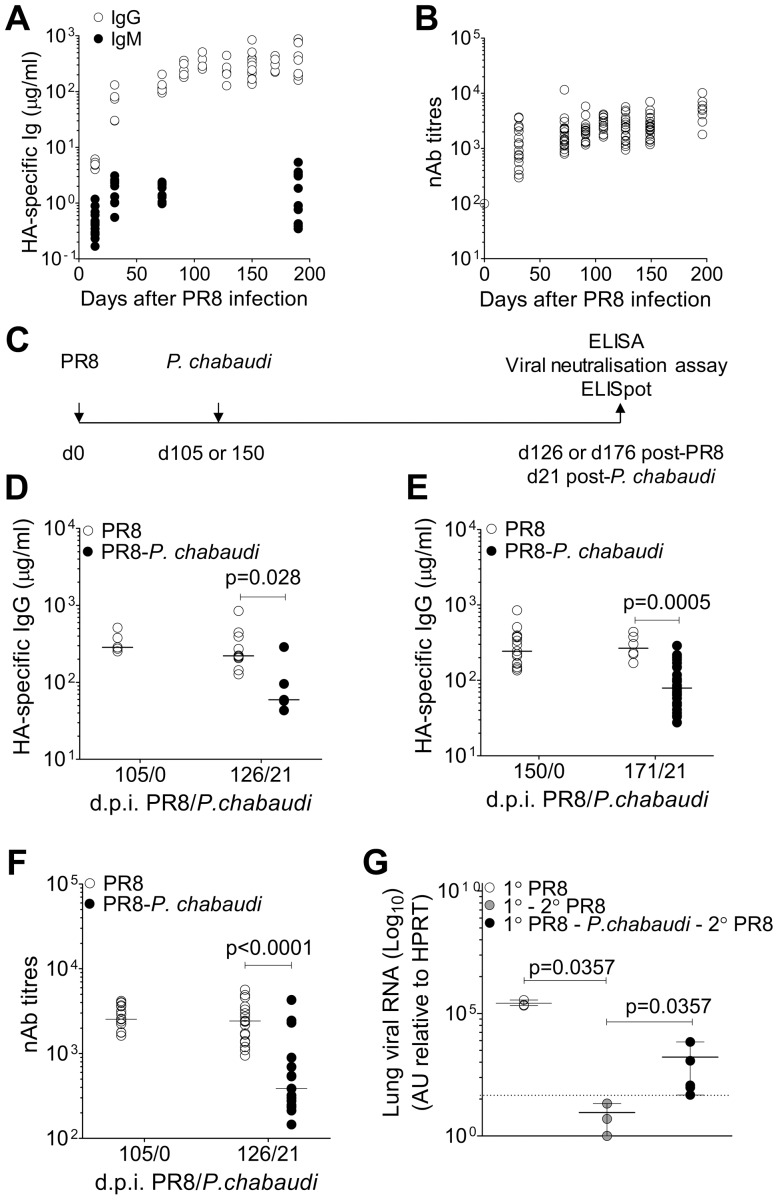
Loss of pre-established Influenza-specific humoral immunity after infection with *P. chabaudi*. 8–10 wk old female BALB/c mice were infected by intranasal instillation of 250 HAU of Influenza A/PR/8/34. **A.** HA-specific IgG (○) (5 mice per time point) and IgM (9 mice per time point) (•) determined by ELISA. **B.** Virus neutralizing Ab titers in PR8-infected mice. Data were obtained from 2 independent experiments each with 10 mice per group (n = 20 per time point). **C.** Schematic representation of experimental design; 8–10 wk old female BALB/c mice were infected by intranasal instillation of 250 HAU of PR8. **D.** 105 or **e**) 150 days after infection with PR8, mice were infected with 10^5^ *P. chabaudi*-infected red blood cells. After 21 days of the *P. chabaudi* infection, HA-specific IgG in PR8-*P. chabaudi*-infected mice (•) and age-matched control PR8-only mice (○) was quantified by ELISA. In figure **D**, n = 5 (d105/0), 10 (○, d126/21) and 5 (•, d126/21). In figure **E**, n = 15 (d150/0), 6 (○, d171/21) and 30 (•, d171/21). **F.** Virus neutralizing Ab titers in PR8-*P. chabaudi*-infected mice (•) and age-matched control PR8-only mice (○). Data were pooled from two independent experiments (n = 17–19 per group). **G.** 8–10 wk old female BALB/c mice were infected by intranasal instillation of 250 HAU of PR8. 150 days later, infected with *P. chabaudi* and drug-cured with chloroquine as described in the Materials and Methods. Six weeks after *P. chabaudi* infection, mice were re-infected with PR8 and viral loads determined after 3 days as described in Materials and Methods. The data were obtained from naïve BALB/c mice (○, n = 3), PR8-immune mice (•, n = 3) and PR8-immune mice infected with *P. chabaudi* (•, n = 5). The dotted line indicates the limit of detection. In Figure **A–D**, data were determined to have an approximately normal distribution (KS normality test, P>0.05) and error bars indicate mean ± S.E. In figures **E–G**, data were determined not to have an approximately normal distribution (KS normality test, P≤0.05), therefore error bars indicate the median ± S.E. and statistical values were calculated using a two-tailed Mann-Whitney test. Non-significant values (P>0.05) are not indicated on the graphs.

BALB/c mice were first infected with PR8 and the kinetics of Ab induction, specific serum Ab concentrations and specific plasma cells and MBCs were quantified at various time points after infection. Intranasal PR8 infection resulted in a gradual increase in serum HA-specific IgG (**Fig. 1A**), which plateaued at a median of approximately 100 µg/ml 80–100 days post infection, and remained stable for a further 100 days. By contrast, HA-specific IgM increased within the first 14 days, but did not increase further over the 200-day period of the experiment (**Figure 1A**). Serum neutralizing Ab (nAb) titers measured by a modified viral neutralization test [16] followed a very similar kinetic to HA-specific IgG Ab response measured by ELISA, peaking approximately 80 day after infection and remaining stable for up to 200 days post-infection (**Figure 1B**)

To determine whether the established anti-PR8 humoral response would be affected by a malaria infection, BALB/c mice were infected with 10^5^ *P. chabaudi* pE 105 or 150 days after intranasal inoculation of PR8 (**Figure 1C**), when the PR8 HA-specific IgG response was stable. Infection with *P. chabaudi* at both time points caused a significant reduction in HA-specific IgG within 21 days after the *P. chabaudi* infection (**Figure 1D–E**). Importantly the loss of HA-specific IgG Abs was accompanied by a substantial decrease in titers of PR8 neutralizing Abs (**Figure 1F**), and consequently there was also a significant loss of anti-viral immunity (**Figure 1G**), as shown by the increased viral titers on day 3 upon re-challenge of these PR8-*P. chabaudi* infected mice with PR8 42 days after *P. chabaudi* infection. Although the cellular immune response to Influenza A virus rechallenge can be highly protective, it is typically delayed in comparison with the immediate protected afforded by pre-existing Abs [17]. Therefore, susceptibility to PR8 re-challenge at this early time-point would be indicative of the loss of PRR-specific humoral immunity after *P. chabaudi* infection.

The loss of PR8-specific Abs was not due to a reduction in half-life of IgG, as neither acute nor chronic *P. chabaudi* infection induced increased clearance of IgG (**Figure S2**). We also established that there was little cross-reactivity of Abs induced by each infection (**Figure S1A–C**), and Abs induced by infection with *P. chabaudi* alone were not able to neutralize PR8 *in vitro* (**Figure S1D**).

Therefore, a *P. chabaudi* infection induced loss of pre-established PR8-specific Abs, which was unrelated to homeostatic regulation of immunoglobulin concentrations.

### Loss of pre-established bone marrow plasma cells during acute infection with *P. chabaudi*

After infection or immunization, serum Ab levels are thought to be maintained by long-lived plasma cells (LLPC) in the bone marrow [3]. Therefore, we investigated whether the reduction of HA-specific Abs, and thus reduced immunity to re-infection with PR8, following a *P. chabaudi* infection could be due to the loss of LLPC.

**Figure 2:**
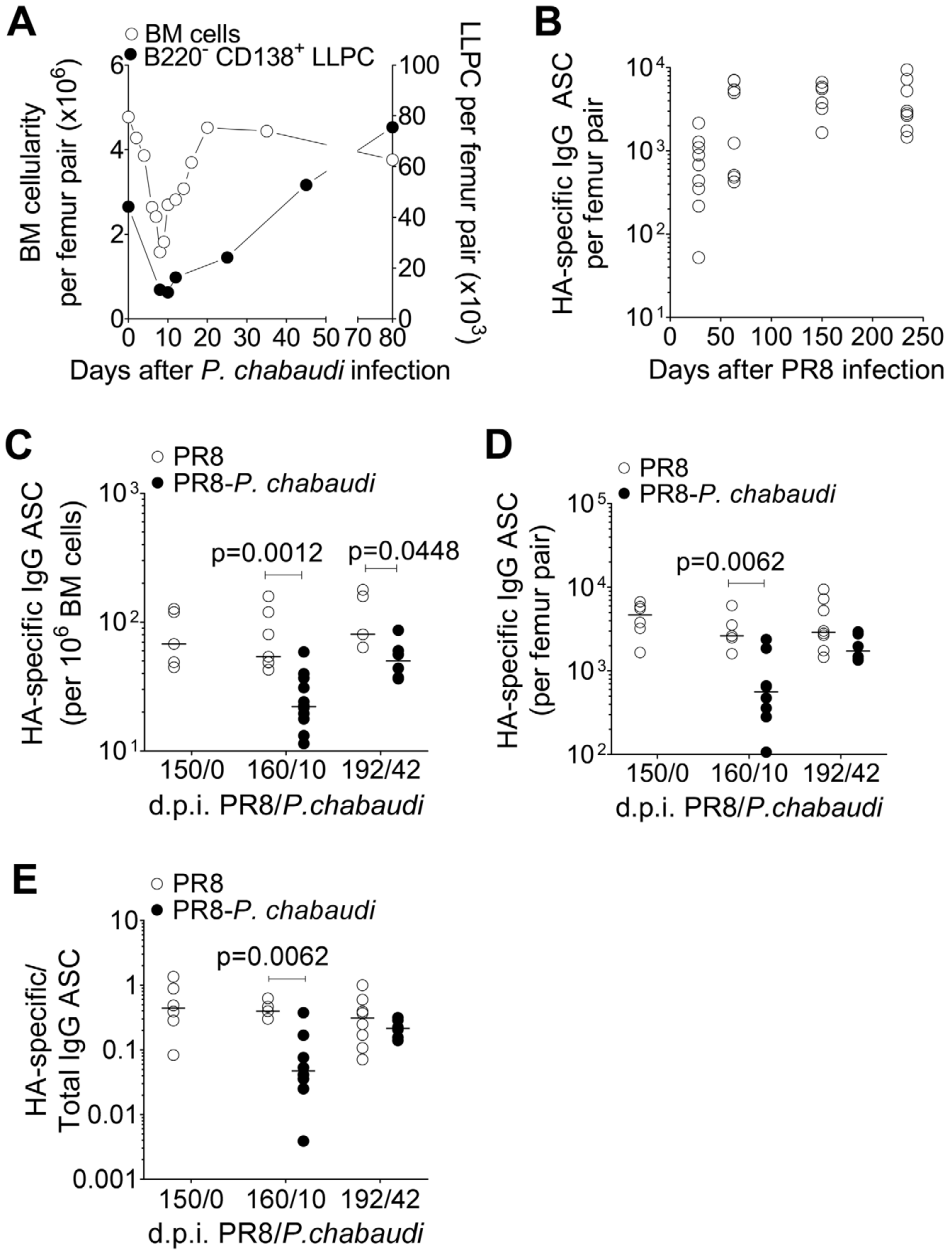
Loss of pre-established Influenza-specific bone marrow plasma cells during acute infection with *P. chabaudi*. Reduction in bone marrow cellularity during acute *P. chabaudi* infection. **A.** Absolute numbers of bone marrow cells per femur pair (○; left *y*-axis) and B220^−^ CD138^+^ long-lived plasma cells (•; right *y*-axis) were quantified over the course of *P. chabaudi* infection. Each point is the median ± S.E. of data obtained from one experiment with 3 mice per time point. **B.** 8–10 wk old female BALB/c mice were infected by intranasal instillation of 250 HAU of Influenza A/PR/8/34. 150 days later, they were infected with *P. chabaudi*. HA-specific IgG ASCs quantified by ELIspot at various times after *P. chabaudi* infection. Graph shows absolute numbers of HA-specific IgG ASC per femur pair [n = 9 (d28); 8 (d63); 6 (d150) and 8 (d234)], and are pooled data from 2–3 independent experiments. **c–e**) Reduction in HA-specific ASCs in the bone marrow in PR8-*P. chabaudi*-infected mice. 8–10 wk old female BALB/c mice were infected by intranasal instillation of 250 HAU of PR8. 150 days later, some mice were infected with *P. chabaudi* as described previously. 10 and 42 days after *P. chabaudi* infection, HA-specific and total IgG antibody-secreting cells (ASCs) in mice infected with PR8 [○; n = 5 (d150/0), 7 (d160/10) and 5 (d192/42)] or PR8-*P. chabaudi* [•; n = 12 (d160/10) and 6 (d192/42)] were quantified using ELISpot. Graph shows numbers of HA-specific IgG ASC **C,** per 10^6^ bone marrow cells; **D,** per femur pair and **E,** expressed as a ratio to total IgG ASC. Data was obtained pooled from 2 independent experiments. In figure **C**, data were determined to have an approximately normal distribution (KS normality test, P>0.05) and error bars indicate mean ± S.E. Statistical values were calculated using a two-tailed Student's *t* test. All non-significant values (P>0.05) are not indicated on the graphs.

First, BALB/c mice were infected with *P. chabaudi* and the absolute number of bone marrow (BM) cells from femur pairs was determined for up to 80 days after *P. chabaudi* infection. In addition, parasitaemia was monitored throughout acute *P. chabaudi* infection. Bone marrow cellularity was reduced by day 8 following the *P. chabaudi* infection (**Figure 2A**), coinciding with the peak of parasitaemia, and then recovered on day 20 as the parasitaemia dropped, and remaining stable for up to 80 days post-infection.

We determined the number of HA-specific Ab-secreting cells (ASC) in the bone marrow as a measure of pre-established LLPC. After a primary PR8 infection, HA-specific ASCs accumulated in the bone marrow and reached a stable number by approximately day 50, and remained at this level for up to 250 days (**Figure 2B**). However, when PR8-immune mice were infected with *P. chabaudi* 150 days later, there was a significant reduction in the number of HA-specific ASC within 21 and for up to 42 days of *P. chabaudi* infection (**Figure 2C** **and** **Figure 2D**). There was a distinct loss of HA-specific ASCs relative to the total IgG ASC compartment in the bone marrow, which remained unchanged (**Figure 2E**) Therefore, infection with *P. chabaudi* resulted in the rapid and sustained loss of pre-established HA-specific ASC in the bone marrow.

### Increased apoptosis of bone marrow long-lived plasma cells during acute *P. chabaudi* infection

The loss of HA-specific ASC from bone marrow during acute P. chabaudi infection could be due to dislocation by competition with migratory plasmablasts, as previously suggested during a secondary immunization of human subjects with tetanus toxoid [18], or by apoptosis of LLPC, as previously described during infection with non-lethal P. yoelii [10] and after immunization with a immunogenic cocktail of antigens [13].

**Figure 3:**
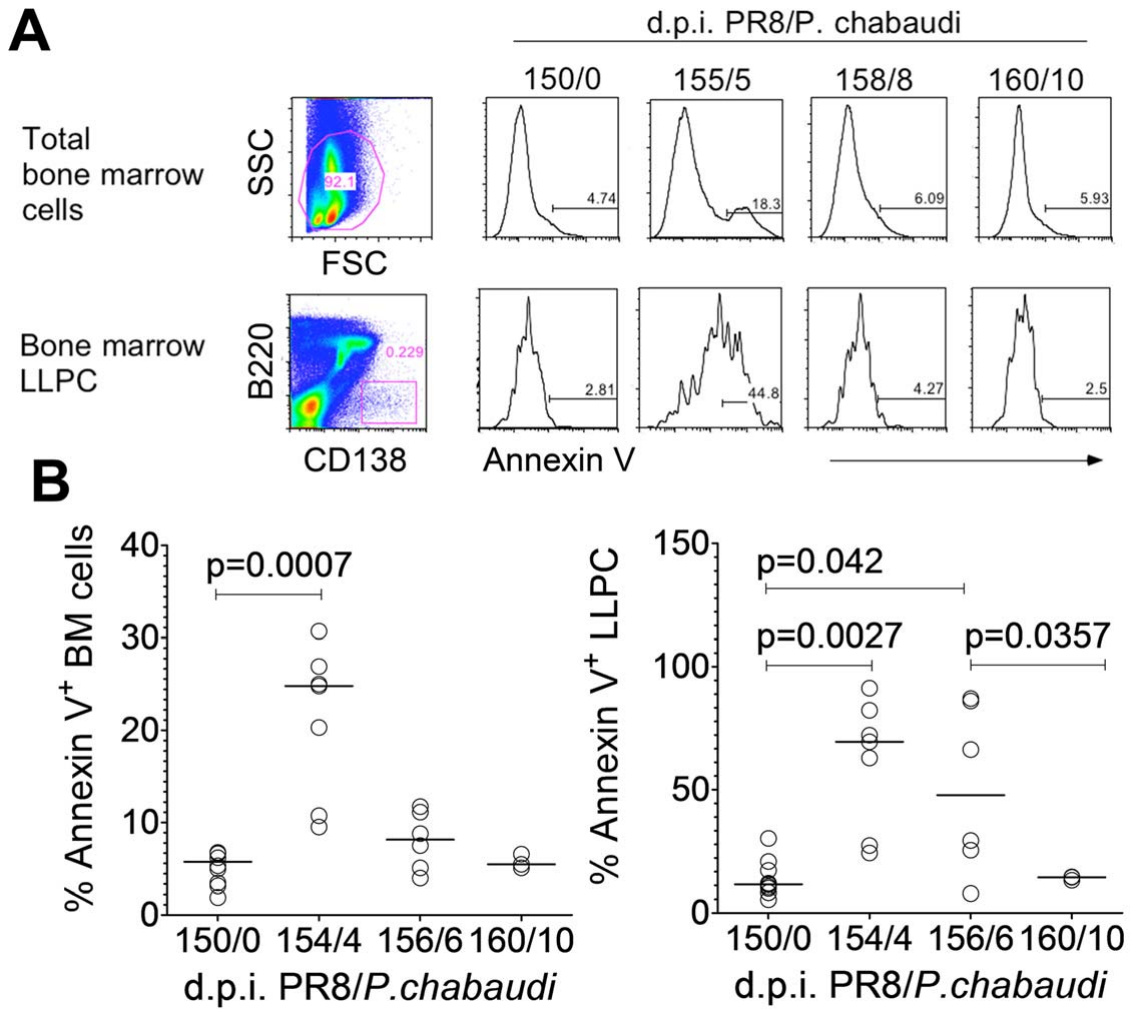
Long-lived plasma cells undergo apoptosis during acute *P. chabaudi* infection. A. Representative flow cytometry plots of total bone marrow cells and bone marrow LLPCs; and relative expression of Annexin V on days 0, 5, 8 and 10 of a *P. chabaudi* infection in BALB/c mice. **B.** Percentage Annexin V expression on total bone marrow cells and bone marrow LLPC on days 0, 4, 6 and 10 of *P. chabaudi infection* (n = 10 (d0); 5 (d4); 6 (d6) and 3 (d10). Data were pooled from two independent experiments.

Newly generated plasma cells (CXCR4^+^ CXCR5^−^ CD19^−^ MHCII^+^) were transiently detected in the blood between days 8 and 12 of a P. chabaudi infection (**Figure S3C**), and migratory plasmablasts (B220^+^ CD138^+^) were present in the bone marrow from day 10 onwards (**Figure S3A and B**). Despite the influx of these plasmablasts, we did not detect any increase in B220^−^ CD138^+^ LLPCs (**Figure S3C**) or HA-specific ASC (**Figure S3D**) above background levels in the blood on days 8 or 10 of P. chabaudi infection, indicating that it was unlikely that pre-established HA-specific ASC were competitively dislocated from bone marrow by migratory plasmablasts, at least not in sufficient frequencies for detection by flow cytometry or HA-specific ELISpot in the blood. By contrast, there was a significant but transient increase in the numbers of Annexin V^+^ apoptotic bone-marrow cells and bone-marrow LLPCs 4 days after a P. chabaudi infection (**Figure 3A–B**).

### Fcγ Receptors are important for mediating apoptosis of LLPC after a *P. chabaudi* infection

FcγRIIB expressed on LLPCs has been previously implicated in homeostatic regulation of the LLPC niche, in a cell-intrinsic manner, during immune responses by inducing apoptosis of LLPC after ligation by elevated concentrations of immune complexes [13]. Since we observed dramatically elevated levels of total serum IgG during acute and chronic *P. chabaudi* infection (**Figure S4B**), we investigated whether apoptosis of LLPC during *P. chabaudi* infection could be mediated via immune complex ligation of FcγRs.

**Figure 4:**
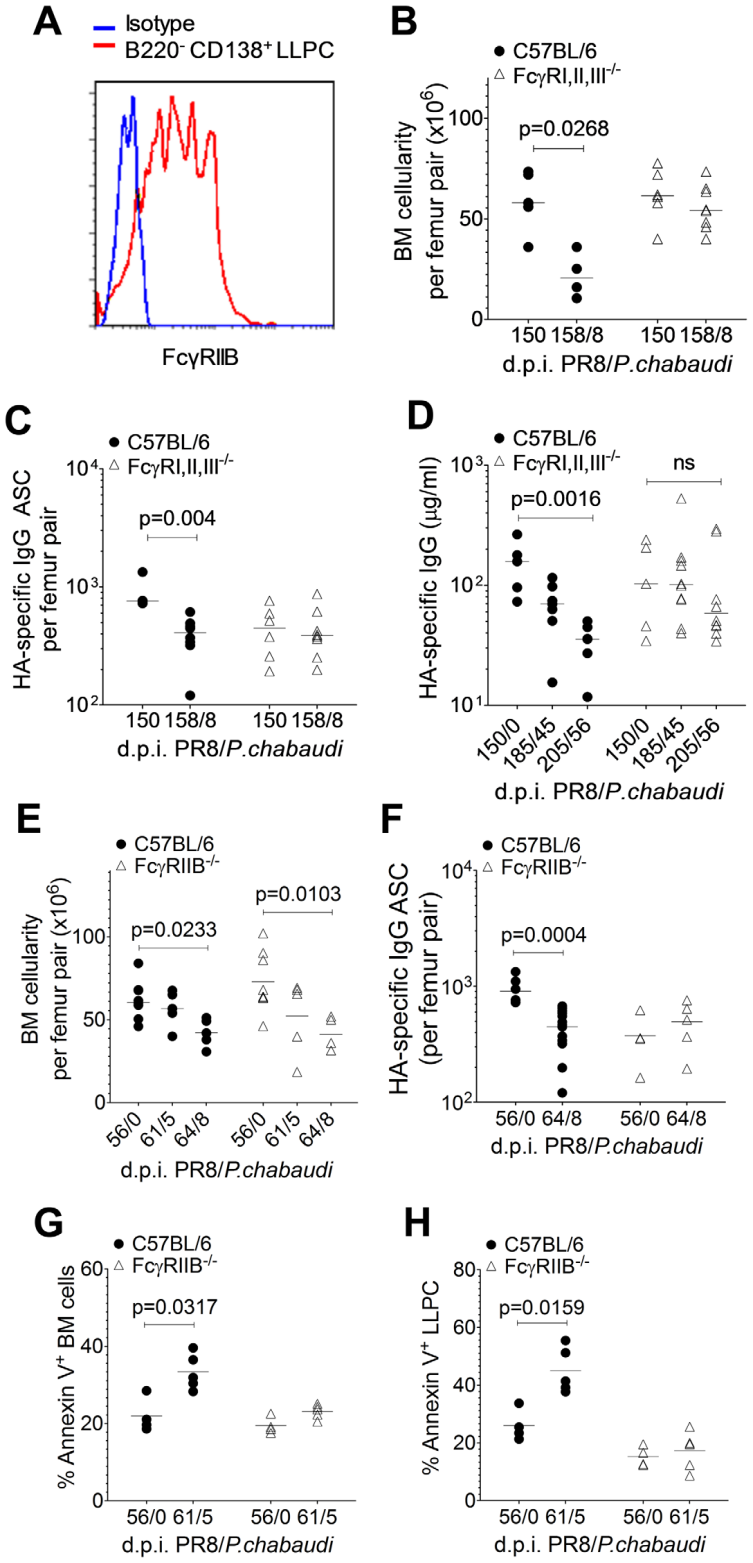
Loss of pre-existing humoral immunity to Influenza A virus is dependent on FcγRIIB. Female C56BL/6 (•) and FcγRI,II,III^−/−^ (Δ) mice aged 8–10 weeks were infected by intranasal instillation of 250 HAU of PR8. 150 days later, mice were infected with *P. chabaudi*. **A.** Representative FACS plot showing the relative MFI of FcγRIIB (CD32) expression on B220^−^ CD138^+^ bone marrow LLPCs compared to an isotype control. **B** and **C** 8 days after *P. chabaudi* infection, bone marrow from femur pairs was obtained. **B.** Total bone marrow cellularity was determined and **C,** HA-specific ASCs were quantified using ELISpot [FcγRI,II,III^−/−^ (Δ): n = 6 (d150/0) and 8 (d158/8); C56BL/6 (•): n = 3 (d150/0) and 8 (d158/8)]. Data were pooled from two independent experiments. **D.** Venous blood was obtained from C56BL/6 and FcγRI,II,III^−/−^ mice on days 0, 35 and 56 of *P. chabaudi* infection and processed for serum. HA-specific IgG antibodies were quantified by ELISA. Data was obtained from one experiment [FcγRI,II,III^−/−^ (Δ): n = 5 (d150/0), 10 (d185/35) and 10 (d196/56); C56BL/6 (•): n = 5 (d150/0), 7 (d185/35) and 7 (d196/56)]. **E–H.** 8–10 week old female C56BL/6 (•) and FcγRIIB^−/−^ (Δ) mice were infected by intranasal instillation of 250 HAU of PR8. 56 days later, mice were infected with *P. chabaudi* as previously described. **E.** 5 and 8 days after *P. chabaudi* infection, bone marrow from femur pairs were obtained and total bone marrow cellularity was determined. **F.** 8 days after *P. chabaudi* infection, HA-specific ASCs were quantified using ELISpot. 5 days after *P. chabaudi* infection, % Annexin expression was determined on **G,** total bone marrow cells and **H,** bone marrow LLPCs. Data was obtained from one experiment [FcγRIIB^−/−^ (Δ): n = 4(d56/0), 5(d61/5) and 5(d64/8); C56BL/6 (•): n = 3(d56/0), 5(d61/5) and 5(d64/8)]. Error bars indicate the median ± S.E.M. and statistical values were calculated using a two-tailed Mann-Whitney test. Non-significant values (P>0.05) are not indicated on the graphs.

Bone marrow LLPCs induced by PR8 infection expressed FcγRIIB (**Figure 4A**). Using C56BL/6 mice lacking either FcγRIIB (FcγIIB^−/−^) or FcγRI,II, and IIIa (FcγRI,II,III^−/−^), we asked whether HA-specific Abs and HA-specific ASCs were maintained after a *P. chabaudi* infection in the absence of these Fcγ receptors. We established that there was a similar loss of HA-specific antibody and ASC in PR8/*P. chabaudi* infected wild-type C56BL/6 mice compared with BALB/c mice (data not shown), and that the course of infection of *P. chabaudi* were similar in FcγRI,II,III^−/−^ and FcγRIIB^−/−^ mice compared to those of wild-type C56BL/6 (**Figure S5A–C**) and [19].

Despite similar peak parasitaemias, there was no loss of either total bone marrow cellularity (**Figure 4B**) or HA-specific ASCs (**Figure 4C**) on day 8 of *P. chabaudi* infection in FcγRI,II,III^−/−^ mice compared with the significant loss in C56BL/6 mice. In line with this, FcγRI,II,III^−/−^ mice retained their pre-established levels of HA-specific serum IgG for up to 56 days after *P. chabaudi* infection (**Figure 4D**), whereas there was a significant drop in Ab levels in wild-type C56BL/6 mice at this time. Interestingly, although there was no reduction in total bone marrow cellularity in FcγRI,II,III^−/−^ mice after a *P. chabaudi* infection, there was a significant loss of total bone marrow cellularity in FcγRIIB^−/−^ mice infected with *P. chabaudi* (**Figure 4E**), suggesting that the loss of LLPC and the loss of other cells may be via engagement of different Fc receptors. Importantly, there was no loss of HA-specific ASCs in FcγRIIB^−/−^ mice (**Figure 4F**), strongly implicating FcγRIIB as the crucial factor in the maintenance of pre-established LLPCs. In line with this, whilst a substantial fraction of total bone marrow cells and bone marrow LLPC in C56BL/6 mice infected with *P. chabaudi* was apoptotic and expressed Annexin V, there was no change in Annexin V expression levels in infected FcγRIIB^−/−^ mice at all (**Figures 4G and 4H**). Ligation of the FcγRIIB, therefore, is an important mechanism of loss of pre-established HA-specific bone marrow ASCs and serum Abs during *P. chabaudi* infection.

### Re-establishment of HA-specific IgG and HA-specific ASCs to pre-established levels at long-term time points of *P. chabaudi* infection

**Figure 5:**
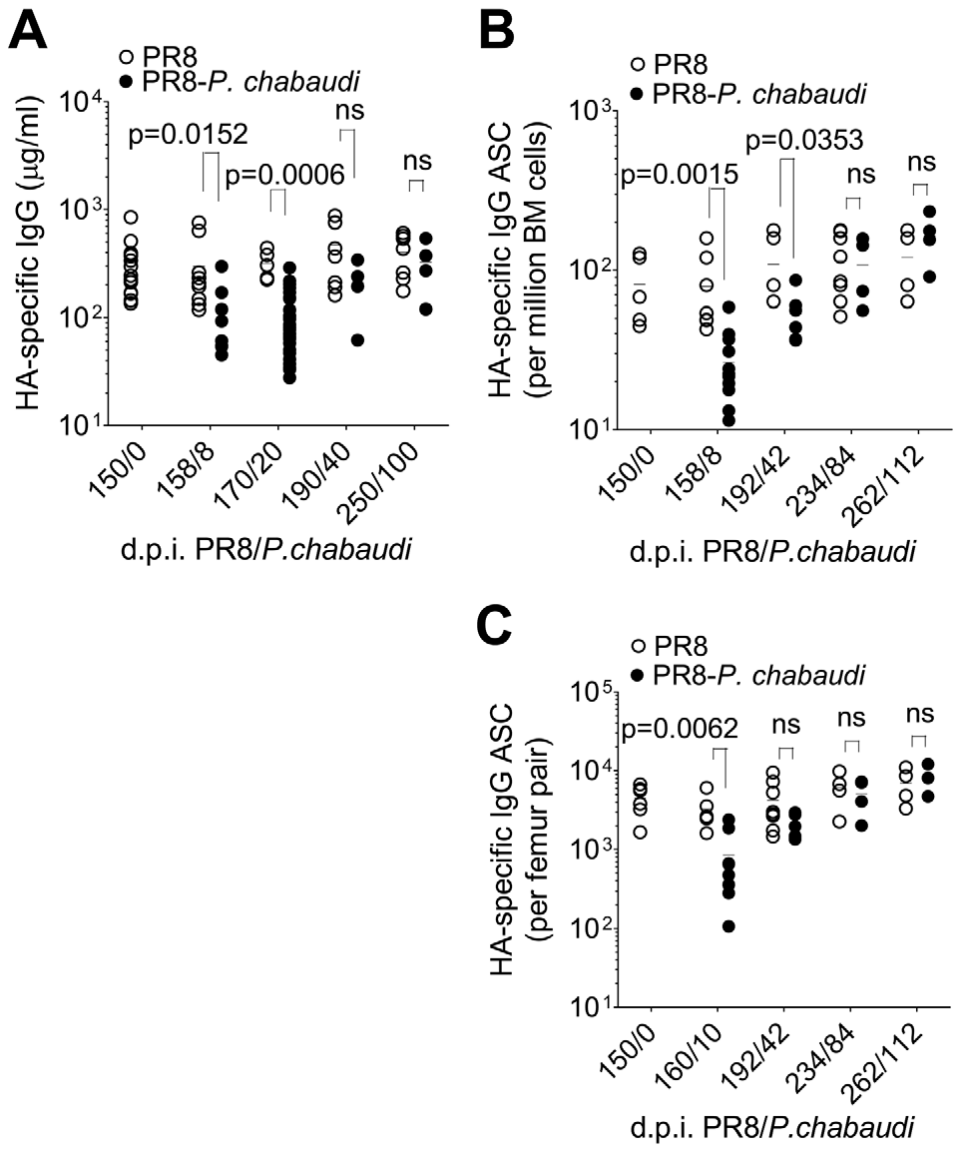
Return of HA-specific IgG and HA-specific IgG ASC to bone marrow at late time points after *P. chabaudi* infection. 150 days after PR8 infection, mice were infected with *P. chabaudi*, **A.** HA-specific IgG quantified by ELISA in PR8-*P. chabaudi*-infected mice [(•); n = 9(d150/0), 5(d158/8), 5(d170/20), 8(d190/40) and 7(d250/100)] and PR8-only controls [(○); n = 9(d150/0), 8(d158/8), 30(d170/20), 4(d190/40) and 4(d250/100)]. **B** and **C.** HA-specific IgG antibody-secreting cells (ASCs) in bone marrow from femur pairs of in PR8-*P. chabaudi*-infected mice (•) and PR8-only controls (○) [PR8-*P. chabaudi* n = 5(d150/0), 12(d158/8), 6(d192/42), 4(d234/84) and 4(262/112); PR8 only 5(d150/0), 7(d158/8), 5(d192/42), 8(d234/84) and 4(262/112)]. The data were pooled from 2 independent experiments and expressed **B**, per million bone marrow cells or **C**, per femur pair. In figures **A–C**, error bars indicate the median ± S.E. and statistical values were calculated using a two-tailed Mann-Whitney test. Non-significant values (P>0.05) are not indicated on the graphs.

To determine the longer-term effects of this *P. chabaudi* infection on the humoral immune response to PR8, we monitored HA-specific IgG in plasma and numbers of HA-specific ASCs in bone marrow for up to 100 days after *P. chabaudi* infection. To our surprise, we observed a gradual return of HA-specific IgG by day 42 of *P. chabaudi* infection to the previously established levels prior to the *P. chabaudi* infection (**Figure 5A**). Similarly, the numbers of HA-specific ASCs returned to pre-established numbers 84 days after *P. chabaudi* infection, and thereafter remained at this level for up to 110 days after *P. chabaudi* infection (**Figure 5B**), suggesting that HA-specific ASCs are being replenished in the bone marrow, despite no re-infection with PR8. This increase in plasma cells follows a similar kinetic to the restitution of HA-specific Abs in serum.

### B cells are necessary for the maintenance of long-lived serum Ab to PR8

Memory B cells (MBCs) can differentiate into Ab-secreting plasma cells on re-challenge with the specific antigen through the B cell receptor or stimulation of Toll-like receptors (TLRs) [20], [21]. Bystander CD4^+^ T cell help *in vitro* can also stimulate non-specific MBCs to differentiate into PCs, possibly because of an increased availability and upregulation of co-stimulatory molecules and production of Th2 cytokines [21], [22]. We hypothesized that the differentiation of MBCs or other B cell subsets into plasma cells was responsible for the eventual replenishment of bone marrow HA-specific ASCs and thus of serum HA-specific IgG concentrations to pre-established levels before the *P. chabaudi* infection.

To determine whether B cells in general and MBCs in particular can contribute to the maintenance of HA-specific serum IgG after infection with PR8, we selectively depleted B cells, but not LLPCs *in vivo* in PR8-immune hCD20 transgenic mice using the monoclonal anti-hCD20 Ab, 2H7 [23], [24]. This depletion was highly specific for B cells as determined by the surface markers sIgD, CD19 and CD21 (**Figure S6C–D**). A two-week course of 2 mg/wk treatment with the mAb (**Figure S6B**) depleted more than 90% of all B cells in spleen, peripheral blood and lymph nodes, and more than 50% of B cells in bone marrow of PR8-immune hCD20tg mice, but not in their hCD20tg-negative littermates (**Figure S6D**). In line with previous studies [25], [26], treatment with anti-hCD20 mAb did not affect total numbers of CD138^+^ B220^−^ LLPC in the spleen and bone marrow in hCD20tg mice and hCD20tg-negative littermates either in immediately after treatment or 150 days after treatment (**Figure S6E**). Similarly, treatment with anti-hCD20 mAb did not deplete pre-existing GC B cells in the spleen (**Figure S6F**). Instead, numbers of splenic GC B cells were transiently elevated in both hCD20tg and hCD20tg-negative mice immediately after anti-hCD20 mAb administration (**Figure S6F**).

**Figure 6:**
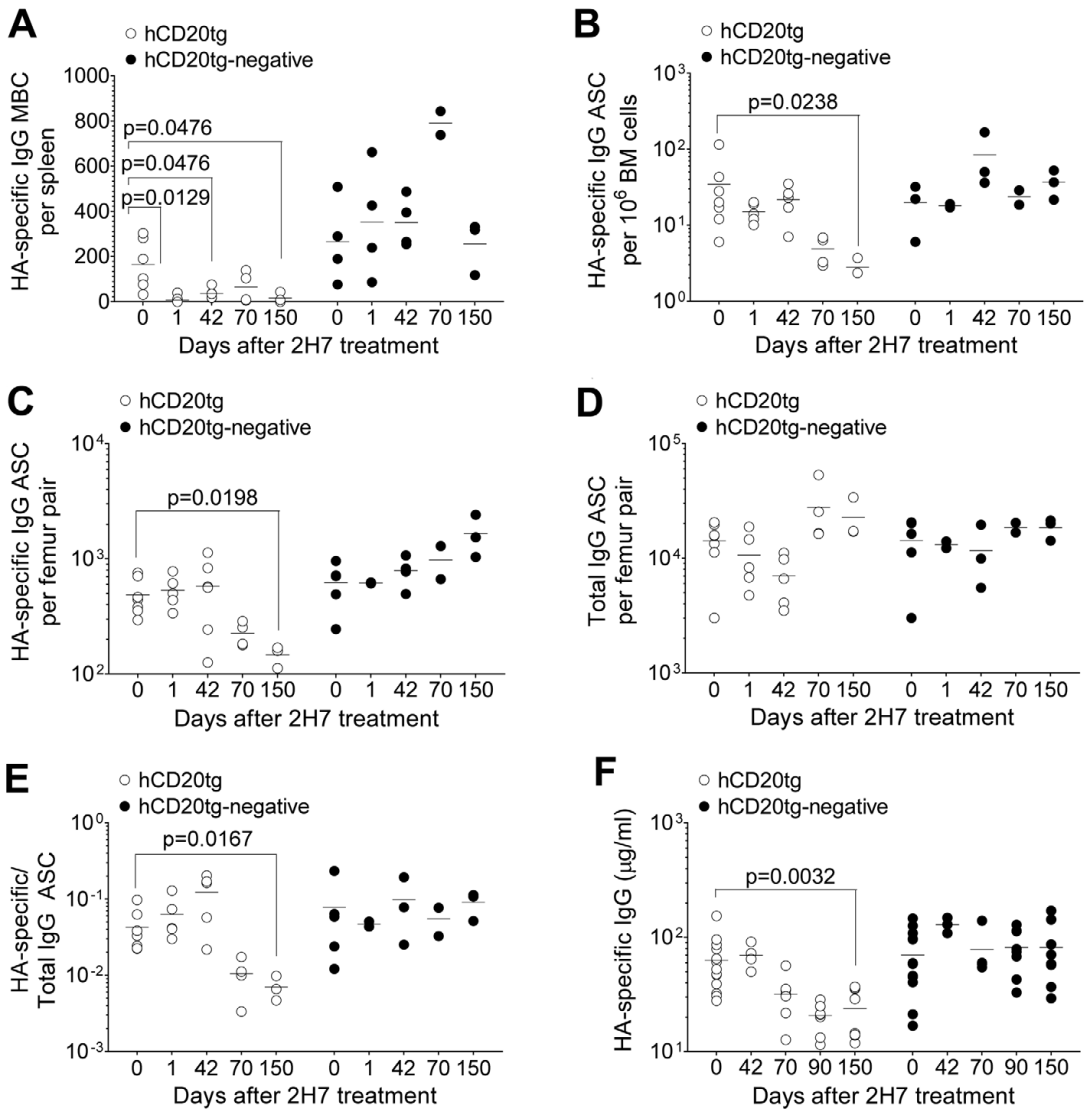
Eventual loss of HA-specific ASCs and HA-specific serum IgG after depletion of HA-specific MBC. 8–10 wk old female hCD20tg and hCD20tg-negative littermates were infected with Influenza A/PR/8/34, and treated with anti-hCD20 mAbs as described in the Materials and Methods. **A.** HA-specific IgG MBCs in spleens from hCD20tg (○) and hCD20tg-negative (•) just before treatment and 1 day, 42 days, 70 days and 150 days post-treatment [hCD20tg: n = 5(d0), 5(d1), 3(d42) and 3(d150); hCD20tg-negative littermates: n = 4(d0), 2(d1), 2(d42) and 3(d150)]. The data are pooled from 3 independent experiments. **B–D.** Femur pairs were obtained from mice and HA-specific and total IgG bone marrow ASCs were quantified using ELISpot in hCD20tg (○) and hCD20tg-negative (•) mice prior to treatment and 1 day, 42 days, 70 days and 150 days post-treatment. [hCD20tg: n = 7(d0), 5(d1), 5(d42) and 3(d150); hCD20tg-negative littermates: n = 5(d0), 2(d1), 2(d42) and 3(d150)]. The graphs show number of HA-specific ASC **B,** per 10^6^ bone marrow cells and **C,** per femur pair; **D,** number of total IgG ASC per femur pair; and **E,** numbers of HA-specific IgG ASC expressed as a ratio to total IgG ASC. The data are pooled from 3 independent experiments. **F.** Serum was obtained and HA-specific serum IgG were quantified in hCD20tg (○) and hCD20tg-negative (•) just before treatment, and 42, 70, 90 and 150 days post-treatment by ELISA [hCD20tg: n = 13(d0), 4(d42), 8(d90) and 8(d150); hCD20tg-negative littermates: n = 13(d0), 3(d42), 8(d90) and 8(d150)]. The data were obtained from 2 experiments. In figures **A–F**, error bars indicate the median ± error and statistical values were calculated using a two-tailed Mann-Whitney test. Non-significant values (P>0.05) are not indicated on the graphs.

The majority of MBCs are thought to reside in the spleen. We therefore quantified HA-specific MBC cells in the spleen with ELIspot as a representative measure of total depletion efficacy. More than 90% of HA-specific MBCs were depleted rapidly from the spleen and this loss remained significant even 150 days after depletion (**Figure 6A**), indicating significant long-term depletion of the vast majority of pre-established HA-specific MBCs. Treatment with anti-hCD20 mAb had no effect on the mean of HA-specific MBCs in hCD20tg-negative littermate controls (**Figure 6A**).

Contrasting the rapid and sustained reduction in HA-specific MBCs, the total MBC niche appeared to be rapidly depleted, but filled up to pre-existing numbers by day 42 (data not shown), presumably with MBC of irrelevant specificities.

In contrast to HA-specific MBCs (**Figure 6A**), HA-specific ASCs in bone marrow were not immediately depleted by anti-hCD20 mAb treatment (**Figure 6B and C**). However, we observed a significant decrease in the number of HA-specific ASCs from 70 days post-depletion onwards in hCD20tg mice, but not in hCD20tg-negative littermates (**Figure 6B and C**). In contrast, there was no change in total IgG ASCs in hCD20tg mice, even 150 days post-depletion (**Figure 6D**). The eventual loss of HA-specific ASCs, but not of total IgG ASCs (**Figure 6E**), suggested that this loss was not a non-specific effect of 2H7 treatment, but perhaps a consequence of the long-term depletion of the HA-specific B cell compartment, which is therefore unable to replenish the HA-specific ASC niche.

Finally, we observed a significant loss of HA-specific IgG only from 70 days post-depletion and for up to 150 days post-depletion (**Figure 6F**), without any change in total serum IgG in hCD20tg mice (data not shown). This eventual loss of HA-specific ASC and HA-specific serum IgG at later time-points after depletion of HA-specific B cells demonstrates the importance of a complete HA-specific B cell compartment to maintain numbers of HA-specific ASC and HA-specific IgG in mice. The kinetics of the loss of HA-specific ASC and HA-specific Ab were very similar. From these data (**Figure 6C and F**), in the absence of a HA-specific B cell compartment, the half-life of HA-specific ASC was calculated to be approximately 72 days, whilst the half-life of serum HA-specific Abs is approximately 86 days.

The requirement for HA-specific B cells in maintaining long-term HA-specific ASCs and Abs strongly suggests that HA-specific B cells are very likely to be responsible for the eventual return of HA-specific ASCs, and therefore serum HA-specific Ab at late time points of *P. chabaudi* infection.

## Discussion

We have used a mouse model to examine the requirements for maintenance of long-term humoral immunity to Influenza A virus, a virus that induces life-long humoral immunity in humans [2]. We show that serum levels of Influenza A virus-neutralizing Abs are maintained by continuous conversion of Influenza A virus-specific B cells into antibody-secreting LLPCs under steady-state conditions. A single malaria episode significantly dysregulates this maintenance of Influenza A virus-neutralizing Abs: a *P. chabaudi* infection, initiated after the B cell and antibody responses to Influenza A virus reach a stable plateau, results in the loss of pre-established serum Abs and plasma cells specific to Influenza A virus and in increased susceptibility to Influenza A virus re-infection. The loss of LLPC and the reduction in Abs is mediated via an FcγRIIB-dependent mechanism resulting in their apoptosis. However, continuous conversion of Influenza A virus-specific B cells into LLPCs following *P. chabaudi* infection eventually replenishes the LLPC pool and restores humoral immunity to Influenza A virus, highlighting that this arm of adaptive immunity can withstand considerable homeostatic disruption.

Homeostatic regulation of LLPC occurs as they compete for space in survival niches, supported by intrinsic and extrinsic survival resources [27]. The size of the LLPC niche in the bone marrow is finite [4], [28], [29] and has to accommodate LLPCs with specificities against different infections over time [4]. LLPCs may therefore be lost from their niches by competition from new migrating plasmablasts generated by heterologous infections [4], [18]. During each new immune response, some of the pre-established LLPCs are assumed to be removed [4], [30]. If the numbers of newly migrating plasma cells are sufficiently large to reduce the number of established antigen-specific LLPCs to below the threshold required to sustain enough specific serum Abs to neutralize re-infection, the host may effectively lose protective immunity to that pathogen. Loss of Influenza A virus-specific ASCs and concomitant loss of protective immunity to Influenza A virus re-infection following *P. chabaudi* infection, in the face of a total IgG ASC pool which did not change during *P. chabaudi* infection, strongly suggests that such a mechanism operates in this context. Although our analysis took place on day 3 post-rechallenge, which was heavily reliant on pre-existing Abs for protection [17], we cannot formally exclude the potential contribution of cellular immunity. However, unlike the finite LLPC compartment, it is thought that the memory CD8^+^ T cell compartment is expandable [31] and therefore perhaps not as susceptible to stochastically-determined attrition as the LLPC pool is.

Several mechanisms have been put forward to explain loss of LLPCs from bone marrow as a result of subsequent infection or immunization. As suggested for developing B cells in *Trypanosoma brucei* infections of mice [32], loss of expression of CXCL12 on LLPCs, which is required for their retention in BM [33], could result in displacement from the BM and subsequent cell death. Although we did not investigate levels of CXCL12 expression on LLPCs, we found no evidence of displacement of LLPCs or HA-specific ASCs into the blood, suggesting that this may not explain the loss of ASCs in this *P. chabaudi* infection. Rather, our data support the idea that plasma cells undergo apoptosis *in situ* in the bone marrow, in agreement with previous studies in *P. yoelii* infections, in which caspase-3-dependent apoptosis of MBCs and LLPCs was evident [10].

Apoptosis of LLPCs following injection of an immunogenic cocktail of antigens has been suggested to result from binding of immune complexes to the inhibitory FcγRIIB expressed on B cells and plasma cells [13]. A cell-intrinsic role for FcγRIIB in LLPC apoptosis was demonstrated in adoptive transfer studies of wild-type and FcγRIIB-deficient immune splenocytes, which were then differentiated into LLPCs upon secondary challenge [13]. Early studies using *P. chabaudi*-infected mice also implicated immune complexes of lipoproteins and IgG in inhibiting Ab secretion from ASCs *in vitro* [14]. Here, we show that loss of Influenza A virus-specific LLPCs is also dependent on FcγRIIB, as mice lacking this receptor did not lose pre-established HA-specific ASCs and their LLPCs did not undergo apoptosis following a *P. chabaudi* infection. Although, a cell-intrinsic role of FcγRIIB in LLPC loss, as previously demonstrated [13], was not investigated in this study, it is likely that the observed loss of LPPCs is brought about by FcγRIIB-dependent apoptosis, triggered by extensive hypergammaglobulinemia and the generation of immune complexes that occur during acute *P. chabaudi* infection [8], [34].

Surprisingly, in this study we found that Influenza A virus-specific Abs eventually returned to the levels observed before the *P. chabaudi* infections, suggesting that specific LLPCs were being replenished despite the fact that the Influenza A virus infection had been eliminated several months previously. One explanation for this is that Influenza A virus-specific B cells have been reactivated, differentiating into PCs and repopulating the Influenza A virus-specific LLPC niche. Indeed, we could show that B cell depletion in Influenza A virus-immune hCD20-transgenic mice resulted in specific depletion of Influenza A virus-specific B cells and eventual loss of LLPCs and Influenza A virus-neutralizing Abs. In contrast, the total IgG ASC population in the bone marrow, the major LLPC reservoir, was not affected by anti-hCD20 mediated depletion, indicating that the eventual loss of HA-specific ASCs and HA-specific IgG was due to a depleted HA-specific B cell pool. Hence our data strongly support a mechanism whereby Influenza A virus-specific B cells contribute to the continuous replenishment of Influenza A virus-specific bone marrow plasma cells and serum Abs.

Generation of Influenza A virus-specific ASCs may also originate either from the recruitment of new naïve B cells into a chronic response, an ongoing low-level GC reaction or reactivation of MBCs. However, the GC B cell population was unaffected during B cell depletion in hCD20 transgenic mice, and the naïve B cell population, as well as the total MBC population was relatively quickly restored following cessation of anti-hCD20 treatment. These two populations could in principle restore Influenza A virus-specific ASCs. However, Influenza A virus-specific serum Abs showed very little recovery following anti-hCD20 treatment, as did the Influenza A virus-specific MBC population. This observation strongly suggests that the continuous seeding of the LLPC pool and replenishment following *P. chabaudi* infection are mediated by MBCs.

MBCs have a variety of properties, which enable them to maintain serum Abs even in the absence of re-infection. MBCs can survive independently of antigen stimulation and in the absence of mitosis [35], are intrinsically programmed for faster signaling and self-renewal [36], and have been documented to re-circulate for up to 90 years after the last known infection [2], [37]. Furthermore, they are unique from other B cell subsets and LLPCs in their independence of the cytokines BAFF and APRIL for their survival [38] and have their own specialized niches like the spleen [39], although the properties of these niches are not well characterized.

While MBCs do not spontaneously differentiate into ASCs, MBCs have a higher propensity to differentiate into PCs than naïve B cells upon activation [21], [40]. MBCs differentiate into plasma cells upon antigenic stimulation and they have the potential to react to a wider range of pathogenic epitopes than the Abs produced by LLPCs, due to their lower-affinity, more polyreactive B cell receptors (BCRs) [41], [42], meaning that both homologous antigen and cross-reactive stimulation can stimulate the MBC B cell receptor. In addition, human and mouse MBCs can differentiate *in vitro* into plasma cells upon non-BCR-mediated, non-specific Toll-like receptor (TLR) stimulation [22], [40]. Therefore there are a number of ways in which MBCs can maintain serum Abs. Over time, MBCs can continually differentiate into ASCs whenever the host encounters homologous re-infection, cross-reactive heterologous infections, or in any inflammatory context with TLR ligands and bystander T cell help, and thus frequently boost serum Ab titers, and/or replenish the LLPC niche [22].

Although antigen-independent MBC conversion to LLPC has been suggested by studies in humans and *in vitro* [22], evidence that non-specific TLR or cytokine-mediated reactivation of MBC occurs *in vivo* in mice is currently lacking [40]. In contrast, mouse studies have demonstrated that persistence of viral antigens following Influenza A virus infection can be very long, spanning weeks or months [43]. It is therefore likely that reactivation of Influenza A virus-specific MBCs and continuous generation of LLPCs is a consequence of antigen retention. Further supporting this notion, MBCs have been shown to reconstitute humoral immunity to cytomegalovirus upon adoptive transfer into re-challenged, but not into antigen-free recipients [44], [45], and MBC reactivation in the lung of Influenza A virus re-challenged mice requires the presence of intact viral particles [46]. Therefore, both antigen retention and continuous generation of virus-specific ASCs may be seen as part of a robust mechanism to ensure both long-term maintenance of Influenza A virus-neutralizing serum Abs and recovery following episodes of attrition.

These reports indicate that there is a strong biological basis for the importance of MBCs, not just in the anamnestic response, but also in the general maintenance of long-lived serum Abs in the absence of re-infection. However these findings are seemingly at odds with the notion that serum Abs are maintained only by LLPCs, which was inferred by previous B cell depletion studies in mice [25], [26]. The latter studies suggested that the LLPCs generated by protein immunization and acute infection with LCMV (Armstrong) [3] are capable of surviving and maintaining pre-established serum Ab titers for long periods of time, despite MBC depletion by monoclonal Abs or irradiation. Our findings suggest that maintenance of long-term humoral immunity exclusively by LLPCs, in the absence of input from B cells, might not be a universal feature of all viral infections. The degree of reliance of the LLPC pool and consequently of protective immunity on continuous conversion of B cells into LLPCs is likely dependent on the size of the virus-specific LLPC pool. A large LLPC pool will require less B cell input before its size is reduced below the critical threshold for protection. In contrast, a small LLPC pool will not be able to resist attrition without continuous B cell input.

Our data have implications for the longevity of protective efficacy of vaccinations in malaria-endemic countries. Field data in humans on the impact of malaria infection on pre-existing immunity are relatively limited. The efficacy of childhood vaccination is reduced in malaria-endemic countries such as Nigeria or The Gambia as compared to non-endemic areas [47], [48], although the precise underlying reasons are not clear. Furthermore, there are documented outbreaks of infectious diseases, such as polio, despite a high level of vaccination coverage in the same areas [49]. However, as the majority of vaccine efficacy studies are carried out in very young infants and children, they might be confounded by a number of factors, such as the immaturity of the immune system [50] and high pre-existing titers of maternal Abs, which inhibit the development of MBCs and LLPCs. It has been documented that a co-infection with *P. falciparum* (i.e. detectable parasitaemia) suppresses the development of vaccine-induced immune responses [47], [51], although there are also reports that overall vaccine-induced immunity has not been affected in malaria-endemic countries [52]–[55]. Similar studies in farm animals have shown that infection with African trypanosomes significantly reduced the efficacy of several commercial vaccines [56]–[60]; however almost all these studies were done with vaccinations given during the time of Trypanosome infection and so do not provide answers to whether a parasite infection caused a loss of pre-established immunity. There is a dearth of investigations into the longevity of pre-established immunity in older age groups living in or moving into malaria-endemic countries where the parasite may have the ability to abrogate pre-established immunity and render the host susceptible to secondary infection. This would be extremely relevant in the context of multiple vaccination programs in malaria-endemic countries.

## Materials and Methods

### Ethics statement

All animal experiments were approved by the ethical committee of the NIMR, and conducted according to local guidelines and UK Home Office regulations under the Animals Scientific Procedures Act 1986 (ASPA) and the authority of Project Licenses PPL 80/2236 and PPL 80/2358.

### Mice

Inbred C56BL/6 and BALB/c mice were originally obtained from the Jackson Laboratory (Bar Harbor, ME) and subsequently bred and maintained in a specific pathogen-free (SPF) unit at the MRC National Institute for Medical Research (NIMR, London) animal facilities for over 30 years. hCD20tg BALB/c [24], Rag2^−/−^ BALB/c [61], FcγRIIB-deficient (FcγRIIB^−/−^) C56BL/6 mice [62] and FcγRI-, II- and III-deficient (FcγRI,II,III^−/−^) C56BL/6 mice [63] were backcrossed for at least 7 generations onto the NIMR inbred mice and used with age-matched BALB/c and C56BL/6 controls. Experiments were performed using 8–15 week old female mice of each strain. Animal experiments were performed in accordance with the UK National guidelines (Scientific Procedures) Act 1986 under license approved by the British Home Office and the NIMR Institute Ethical Review Panel.

For induction of non-lethal Influenza A virus infection, mice were infected by instillation of the A/Puerto Rico/8/34 strain of (H1N1) Influenza A virus (PR8) into their nasal cavities without anesthesia. For sub-lethal infection, mice were given light inhalation anesthesia with isoflorane before intranasal instillation with PR8 and allowed to recover. Infection with *P. chabaudi chabaudi* (AS) (*P. chabaudi*) was initiated 105 or 150 days after PR8 infection by intraperitoneal (i.p.) injection of 10^5^ iRBCs. Parasitaemia was determined by examination of Giemsa-stained thin blood smears. Chloroquine (CQ) for injection was prepared fresh from chloroquine diphosphate salt (Sigma) for drug-mediated elimination of parasites [8]. The treatment protocol was 10 daily i. p. injections of 40 mg/kg of CQ dissolved in sterile 0.9% saline. The efficacy of the drug treatment was verified by sub-inoculation of blood from the drug treated mice into immune compromised RAG2^−/−^ recipient mice and analyses of thin blood films from the RAG2^−/−^ recipient mice for the next 10 days. The mAb recognizing hCD20, 2H7 [24], was used for B cell depletion. 2H7 was purified from hybridoma culture supernatants and was endotoxin tested with Pyrotell (Associates of Cape Cod) and found to be present at a level of 0.3–0.6 EU/1 ml. Mice were injected i. p. with 2 mg/week of 2H7 in sterile 0.9% saline for 2 weeks.

### Parasites

*P. chabaudi* (AS) parasites were cloned and maintained at the NIMR, London [64], from an original isolate provided by Professor David Walliker (University of Edinburgh). Cryopreserved parasite stabilates were used for initiating infections in the animals in the manner previously described [65]. Briefly, stabilates were thawed from liquid nitrogen, diluted 1∶1 with 0.9% saline and injected i. p. into BALB/c mice. These parasites were passaged up to four times in mice by i. p. injection of 10^6^, 10^5^ or 10^4^ iRBC per mouse diluted in 100 µl of Kreb's glucose saline. The number of iRBC was calculated by determining the percentage of parasitaemia on thin blood films using 20% Giemsa stain (VWR) and assuming a RBC density of 2.5×10^9^/ml in peripheral venous blood. Experimental mice were infected using iRBC taken from one of the passage mice before the peak of parasitaemia. Each experimental mouse received an i. p. injection of 10^5^ iRBC diluted in 100 µl of Kreb's glucose saline.

### SDS-PAGE and western blotting

Bromelain-digested PR8 haemagglutinin (HA) was prepared as previously described [66]. *P. chabaudi*-iRBC were solubilized in Triton X-100 and SDS buffer in the presence of protease inhibitors [67]. Proteins were resolved by electrophoresis through NuPAGE 4–12% acrylamide gels in MES buffer (Invitrogen) under reducing conditions. Markers were the broad-range pre-stained protein standard Seeblue2 (Invitrogen). Proteins were transferred onto Hybond C extra nitrocellulose membrane (Amersham Pharmacia), as described previously [68]. Specific proteins were detected using (i) unpurified sera from influenza immune mice or (ii) unpurified sera from *P. chabaudi* infected mice, followed by Alexa 680-conjugated goat anti-mouse IgG (1∶15,000) (Licor Biosciences) and revealed by scanning the membranes with the Odyssey scanner (Licor Biosciences) using 680EX nm/700EM nm filter settings. Plasma from uninfected mice was used as a negative control.

### Flow cytometry

For surface staining, 5×10^6^ erythrolyzed and washed cells in 50 µl of FACS buffer (PBS, 2% FCS, 0.05% NaN_3_) were stained with prepared antibody multi-mixes (obtained from eBioscience, BD Pharmingen or BioLegend) in the presence of Fc receptor blocking antibody (clone 24G.2) to prevent non-specific binding of Fc receptors. Cells were incubated at 4°C for 40 minutes. Stained cells were washed two times with 200 µl of FACS buffer. For biotinylated antibodies, streptavidin-conjugated fluorochromes were added at a dilution of 1/100 and further incubated for 10 minutes at room temperature and then washed 3 times with FACS buffer. Cells were acquired using the CyAn ADP flow cytometer (Beckman Coulter) within 2 hours. Data were analyzed using FlowJo (Tree Star, Inc). Flowjo was used for graphical representation. For Annexin V staining, after surface staining was completed, cells were washed 2 times and resuspended in Annexin V Binding Buffer (BioLegend) at a concentration of 1×10^6^ cells/ml. 100 µl of the cell suspension was filtered through a 0.2 µm filter into polypropylene FACS tubes (BD) and 5 µl of Annexin V-Pacific Blue (BioLegend) was added. Cells were incubated for 15 minutes at room temperature in the dark. 400 µl of Annexin V Binding Buffer was added to the tube just prior to analysis.

### Neutralization assay

Serum titers of PR8 neutralizing antibodies were measured as previously described [16]. Sera were collected at indicated time points after PR8 infection, heat-inactivated for 10 minutes at 56°C, and tested using a modified Madin-Darby canine kidney (MDCK)-based assay. Serial dilutions of the sera were added to monolayers of MDCK cells in 96-well plates, which subsequently were infected with a 95% tissue culture-infective dose of PR8. MDCK cell viability was measured with an Alamar blue-based assay 3 days after infection. Cultures were pulsed with Alamar blue for 1 to 2 h, and fluorescence was measured with a fluorescence plate reader (TECAN Safire2).

### RNA extraction and cDNA preparation and qRT-PCR

RNA extraction was performed according to the RNeasy mini kit protocol following the manufacturer's protocol (Qiagen cat: 74106). Total RNA was extracted from whole lung tissues using TRI reagent (Sigma-Aldrich) and subsequently was used for cDNA synthesis with the Omniscript reverse transcription (RT) kit (Qiagen). RNA (1 ng) was used as the template, and cDNA synthesis was initiated by a mixture of 1 µM random hexamers and 1 µM of a primer specific to a highly conserved region of the IAV *matrix* gene, as previously described [69], [70]. The following primers were used for the amplification of target transcripts: *Hprt* forward (5′-TTGTATACCTAATCATTATGCCGAG-3′) and reverse (5′-CATCTCGAGCAAGTCTTTCA-3′), *IAV matrix* forward (5′-AAGACCAATCCTGTCACCTCTGA-3′) *and* reverse (5′-CAAAGCGTCTACGCTGCAGTCC-3′). Reaction mixtures were incubated at 37°C for 1 h and terminated by incubating the mixture at 90°C for 5 minutes. Expression of mRNA was determined by quantitative reverse transcription-PCR (qRT-PCR) using a DNA master SYBR green I kit (Roche) and the ABI Prism 7000 detection system (Applied Biosystems). The primers used for the amplification of target transcripts are in the Supplemental Methods [70]. Samples were analyzed in duplicate. The housekeeping gene *Hprt* was used to normalize the critical threshold values for the genes of interest. Levels of IAV *matrix* mRNA are plotted as arbitrary units relative to *Hprt* mRNA levels.

### Enzyme-linked immunosorbant assay (ELISA)

HA-specific IgG or IgM serum antibodies at time points after infection with PR8 and *P. chabaudi* were quantified by ELISA using HA as coating antigen. Results are expressed as µg/ml using an anti-mouse IgG or IgM ELISA (Southern Biotech) and purified Ig (Sigma) as a standard to quantify the amounts of the different isotypes. Amounts of *P. chabaudi*-specific serum antibodies after *P. chabaudi* infection were quantified by ELISA using parasite lysate as a coating antigen using hyperimmune serum from multiply-infected mice as a standard. The amounts of *P. chabaudi*-specific Abs were expressed as arbitrary units (AU) as described [71].

The protocol for determination of the half-life of IgG in mice was adapted from a method previously described [1]. Mice were injected i.p. with 200 µg of anti-TNP mouse IgG2a monoclonal antibodies (Hy1.2; a kind gift of Dr. H–U Weltzien, Max-Planck-Institute for Immunobiology, Freiburg, DE) which was purified from hybridoma culture supernatants as described above for 2H7. Hy1.2 Abs were administered 24 hours or 60 days after infection with 10^5^ iRBC, and into uninfected controls. The concentration of HY1.2 mIgG2a Abs in serum were quantified by ELISA coating with 25 ng/well of TNP-BSA (Biosearch Technologies; diluted in PBS) using purified mouse IgG2a (Sigma) as a standard. Linear regression was used to find the relationship between the logarithm of serum antibody concentration and time since injection. Antibody half-life was then determined using the equation t_1/2_ = (ln 2)/k where k is the decay constant given by the slope of the best fitting linear function.

### Enzyme-linked immunospot assay (ELISpot) for plasma cells and antibody-secreting cells (ASC)

HA-specific plasma cells were quantified by a direct *ex vivo* ELISpot assay as previously described for other antigens [5]. 96-well Multi-screen HA Nitrocellulose filtration plates (Millipore) were coated with 50 µl of 10 µg/ml bromelain-digested PR8 HA diluted in PBS. As a positive control for total IgG secreting cells, some wells on each plate were coated with goat anti-mouse IgG (Invitrogen). The plates were incubated at 4°C overnight, washed twice in PBS, then blocked with 200 µl complete Iscove's medium for 1 h at room temperature. The plates were then washed twice with PBS and cell suspensions added at the following numbers: 1×10^6^, 5×10^5^, 2.5×10^5^ and 1.25×10^5^ per well in 200 µl complete Iscove's medium. The plates were incubated at 37°C, 7% CO_2_ for 5 h, then washed four times in PBS and four times with PBS with 0.1% Tween (PBS-T). 100 µl of goat anti-mouse IgG biotin conjugated antibody (Invitrogen) diluted 1∶1000 in PBS-T containing 1% FCS was added and the plates incubated overnight at 4°C. Plates were washed four times with PBS-T, and 100 µl of streptavidin-alkaline-phosphatase (BD Pharmingen) diluted 1∶8000 in PBS-T containing 1% FCS added and incubated for 1 h in the dark at room temperature, followed by four washes with PBS-T and four washes with PBS. Detection was carried out by adding 100 µl of BCIP/NBT substrate (BioFX) and incubating in the dark until blue spots appeared. The reaction was stopped by thorough washing with cold tap water and air-dried. Plates were analyzed using the ImmunoSpot reader (CTL).

### Enzyme-linked immunospot assay (ELISpot) for memory B cells

HA-specific memory B cells were quantified using a limiting dilution ELISpot technique adapted from [72]. Briefly, 21 replicates of two-fold dilutions of cell suspensions of spleen starting with 1×10^6^ cells per well were made on flat-bottomed 96-well plates (Costar) and cultured for 6 days in 200 µl/well complete IMDM containing a ‘stimulant mastermix’ of 0.4 µg R595 lipopolysaccharide (Alexis Biochemicals), 10^6^ irradiated (1,200 rad) naive splenocytes and 20 µl Concanavalin A supernatant [73] per well. After 6 days, cells were washed in complete IMDM containing 1% FCS, harvested and transferred to pre-coated 96-well Multi-screen Nitrocellulose filtration plates. An *ex-vivo* ELISpot assay for HA-specific and total IgG plasma cell detection performed as described above. Frequencies were determined from the zero-order term of the Poisson distribution, using the Microsoft Excel Trendline option, a straight line of best fit was plotted and values were accepted when r^2^ values were greater than 0.7.

### Statistical analysis

Statistical analysis was performed using GraphPad Prism 5 software. Continuous data, determined to be approximately normally distributed according to the Kolmogorov-Smirnov test (P>0.10), were analyzed using two-sided unpaired Student's *t* test. Where data were not normally distributed, data were analyzed using non-parametric Mann Whitney test. P values≤0.05 were considered significant.

### Accession numbers

- *Cd4* cluster of differentiation 4 antigen [*Mus musculus*]; Gene ID: 12504; Protein ID: NP_038516.1
- *Cd19* CD19 antigen [*Mus musculus*]; Gene ID: 12478; Protein ID: NP_033974.2
- *Cxcr4* chemokine (C-X-C motif) receptor 4 [*Mus musculus*]; Gene ID: 12767; Protein ID: NP_034041.2
- *Cxcr5* chemokine (C-X-C motif) receptor 5 [*Mus musculus*]; Gene ID: 12145; Protein ID: NP_031577.2
- *H2* histocompatibility-2, MHC [*Mus musculus*]; Gene ID: 111364; MGI: 95894
- *HA* haemagglutinin [*Influenza A virus (A/Puerto Rico/8/1934(H1N1))*]; Gene ID: 956529; Protein ID: NP_040980.1
- *Fcgr1* Fc receptor, IgG, high affinity I [*Mus musculus*]; Gene ID: 14129; Protein ID: NP_034316.1
- *Fcgr2b* Fc receptor, IgG, low affinity IIb [*Mus musculus*]; Gene ID: 14130; Protein ID: NP_034317.1
- *Fcgr3* Fc receptor, IgG, low affinity III [*Mus musculus*]; Gene ID: 14131; Protein ID: NP_034318.2
- *MS4A1* membrane-spanning 4-domains, subfamily A, member 1 [*Homo sapiens*]; Gene ID: 931; Protein ID: NP_690605.1
- *Ptprc* protein tyrosine phosphatase, receptor type, C [*Mus musculus*]; Gene ID: 19264; Protein ID: NP_001104786.2
- *Rag1* recombination activating gene 1 [*Mus musculus*]; Gene ID: 19373; Protein ID: NP_033045.2
- *Sdc1* syndecan 1 [*Mus musculus*]; Gene ID: 20969; Protein ID: NP_035649.1

## Supporting Information

Figure S1Determination of cross-reactive antibodies to PR8 and ***P. chabaudi*** **.** Graphs in **A** and **B** plot the mean O.D. (405 nm) values obtained by ELISA after incubating naïve mouse sera (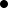), hyperimmune anti-*P. chabaudi* sera (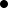), and anti-PR8 sera (○) with either HA or parasite lysate. Results were obtained from one experiment performed in duplicates. Error bars indicate mean ± S.E. In **A**, only PR8 sera contained antibodies binding to PR8 HA. In **B**, *P. chabaudi* sera and, to a lesser extent, PR8 sera, contained antibodies binding to parasite lysate. **C.** Three different preparations of bromelain-digested PR8 HA and *P. chabaudi* lysate were resolved on NuPAGE 12% bis-Tris gels alongside 1× SeeBlue Plus2-prestained standard, electrophorated on Hybond C membrane and probed with normal mouse sera (left panel), hyperimmune *P. chabaudi* sera (middle panel), and PR8 sera (right panel). No cross-reactive antibodies were detected between *P. chabaudi* and PR8 sera. The 46 kDa, fragment of HA was only recognised by PR8 sera. **D.** Graph of the arbitrary virus neutralising Ab titres in naïve mouse sera (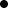), hyperimmune anti-*P. chabaudi* sera (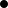), and anti-PR8 sera (○). Reactive serum titres of PR8-neutralising antibodies in the serum was measured using a virus neutralising assay. No neutralising antibodies were detected from 1∶2 serial dilutions of 1∶50–1∶51200 in naïve BALB/c serum and *anti-P. chabaudi* serum. Only PR8 sera were able to neutralise PR8 *in vitro*. Results were obtained from one experiment performed in duplicates. Error bars indicate mean ± S.E.(PDF)

Figure S2
**No difference in half-life of serum antibody during acute or chronic infection with** ***P. chabaudi*** **.** **A.** A schematic of the experiment. 8–10 week old naïve female BALB/c mice were infected with *P. chabaudi*. 24 h or 60 days post-infection, mice were injected i.p. with 200 µg of anti-TNP mIgG2a grown from the Hy1.2 hybridoma. Serum was obtained at various time points after injection. **B.** Concentration of TNP-specific mIgG2a in serum was quantified by ELISA throughout acute *P. chabaudi* infection (d1 post-infection) 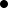) or chronic infection (d60 post-infection) (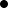) and compared with uninfected age-matched controls (○). Graph showing the mean of data obtained from 2 independent experiments with 5 mice per group. Linear regression was used to find the relationship between the logarithm of serum TNP-specific antibody concentration and time since injection. Antibody half-life was then determined using the equation t_1/2_ = (ln 2)/κ where κ is the decay constant given by the slope of the best fitting linear function.(PDF)

Figure S3
**Movement of B220^+^ CD138^+^ migratory plasmablasts and B220^−^ CD138^+^ LLPC in bone marrow during** ***P. chabaudi*** **infection.** 8–10 wk old female BALB/c mice were infected by intranasal instillation of 250 HAU of PR8. 150 days later, mice were infected with 10^5^ *P. chabaudi* pRBCs. Bone marrow was obtained from femur pairs were obtained at various time points after *P. chabaudi* infection. Migratory plasmablasts (B220^+^ CD138^+^) and long-lived plasma cells (B220^−^ CD138^+^) were quantified using flow cytometry. **A.** Representative FACs plots showing B220 vs CD138 staining of live gated bone marrow cells in naïve BALB/c mice, 150 days after PR8 infection and 12 days after subsequent *P. chabaudi* infection. **B.** Mean and standard error of absolute number per femur pair of migratory plasmablasts (○ B220^+^ CD138^+^) and LLPC (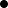 B220^−^ CD138^+^) on days 0, 8, 10, 12, 25, 45 and 75 after *P. chabaudi* infection. Data was obtained from one experiment with 3 mice per time point. Error bars indicate median ± error. **C.** Spleen, PBMC and bone marrow were obtained from femur pairs were obtained on days 0, 7, 8, 10 and 12 after *P. chabaudi* infection. Representative FACS plots of B220 vs. isotype control and B220 vs. CD138 on spleen, PBMC and bone marrow cells 150 days after PR8 infection and 10 days after subsequent *P. chabaudi* infection. Relative mean fluorescence indexes of CXCR4, CXCR5, CD19 and MHC class II were determined by multi-parameter flow cytometry on B220^+^ splenic B cells; B220^+^ CD138^+^ splenic plasmablasts; B220^+^ CD138^+^ migratory plasmablasts in PMBC; B220^+^ CD138^+^ migratory plasmablasts in bone marrow; and B220^−^ CD138^+^ long-lived plasma cells in bone marrow on day 10 of *P. chabaudi* infection. **D.** PBMC were obtained on days 8 and 10 after *P. chabaudi* infection. HA-specific and total IgG antibody-secreting cells (ASCs) in PBMC in mice infected with PR8 (○) or PR8-*P. chabaudi* (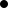) were quantified using ELISpot. Data was obtained from one experiment with 4 mice per time point. Line indicates the mean value. Statistical values were calculated using the Mann-Whitney test.(PDF)

Figure S4
**Development of hyperimmunoglobulinaemia (IgG) during** ***P. chabaudi*** **infection.** 8–10 wk old female BALB/c mice were infected by intranasal instillation of 250 HAU of PR8. 150 days later, mice were infected with 10^5^ *P. chabaudi* pRPBCs i.p. **A.** *P. chabaudi* parasite lysate-specific IgG and **B** total serum IgG at various time points for up to 60 days after *P. chabaudi* infection in PR8-*P. chabaudi*-infected mice (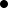) and age-matched control PR8-only mice (○). Each data point represents one mouse from one experiment with 3–5 mice per time point.(PDF)

Figure S5
**Development of HA-specific IgG and parasitaemia in FcγRI,II,III^−/−^ and C57BL/6 mice.** 8–10 wk old female FcγRI,II,III^−/−^ and C57BL/6 were infected by intranasal instillation of 250 HAU of PR8. 150 days later, mice were infected with 10^5^ *P. chabaudi* pRBCs i.p. **A.** Concentration of HA-specific serum IgG in FcγRI,II,III^−/−^ (○) and C57BL/6 (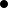) mice on days 28, 56, 84 and 150 after PR8 infection. Line indicates the median value. **B.** % parasitaemia throughout acute *P. chabaudi* infection in FcγRI,II,III^−/−^ (○) and C57BL/6 (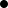) mice, when *P. chabaudi* infection was initiated 150 days after PR8 infection. Graphs indicate the geometric mean ± error of 4 mice per time point. **c**) % parasitaemia at d8 of P. chabaudi infection in FcγRIIB^−/−^ (○) and C57BL/6 (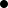) mice, when *P. chabaudi* infection was initiated 56 days after PR8 infection.(PDF)

Figure S6
**Depletion efficacy of treatment with 2H7 mAb in hCD20tg/BALB/c mice.** 8–10 wk old female hCD20tg and hCD20tg-negative littermates were infected by intranasal instillation of 250 HAU of Influenza A/PR/8/34. Venous blood was obtained at various time points up to 150 days post-infection and processed for serum. **A.** The concentration of HA-specific IgG in serum from hCD20tg [○; n = 9(d28), 9(d56), 5(d84) and 13(d150)] and hCD20tg-negative littermates [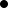; n = 8(d28), 8(d56), 8(d84) and 13(d150)] were quantified by ELISA. Line indicates median values. **B.** Schematic representation of experiment. 8–10 wk old female hCD20tg and hCD20tg-negative littermates and BALB/c WT mice were infected by intranasal instillation of 250 HAU of Influenza A/PR/8/34. 150 days post-infection, mice were treated with 0.5 mg/wk of 2H7/saline i.p every 48 hours for 2 weeks (2 mg/wk). Analysis of efficacy of depletion was done 1 day post-depletion. Assessment of persistence of specific plasma cells and serum antibody was done 42, 84 and 112 days post-depletion by ELISA, flow cytometry and ASC and memory B cell ELIspot. **C.** 1 day post-depletion, spleens were obtained and stained with surface markers as indicated for IgD^+^, CD21^+^ and CD19^+^ B cells. These markers were co-expressed on the majority of B cells. **D.** Graphs show individual data points of the total number of IgD^+^, CD21^+^ and CD19^+^ B cells in spleens before treatment and after anti-hCD20 mAb treatment in hCD20tg [○; n = 7(pre-treatment), 5(d1); 5(d42); 3(d150)] and hCD20tg-negative [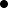; n = 5(pre-treatment), 2(d1); 3(d42); 3(d150)] mice. **E.** Graphs show individual data points of the total number of CD138^+^ B220^−^ LLPCs in spleen and bone marrow before treatment and after anti-hCD20 mAb treatment in hCD20tg [○; n = 7(pre-treatment), 5(d1); 5(d42); 3(d150)] and hCD20tg-negative [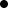; n = 5(pre-treatment), 2(d1); 3(d42); 3(d150)] mice. **F.** Graph shows individual data points of the total number of B220^+^ IgD^−^ GL7^+^ CD38^−^ GC B cells in spleen before treatment and after anti-hCD20 mAb treatment in hCD20tg [○; n = 8(pre-treatment), 5(d1); 3(d150)] and hCD20tg-negative [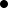; n = 6(pre-treatment), 2(d1); 3(d150)] mice. Line indicates the median value. Statistical values were calculated using the Mann-Whitney test. Statistical values were calculated using the Mann-Whitney test using data pooled from 2 independent experiments. The *n* number of hCD20tg-negative mice was too small for Mann-Whitney test to be performed.(PDF)
