# Supplementary material for: Recovery of an Antiviral Antibody Response following Attrition Caused by Unrelated Infection
Source: PLoS Pathog. 2014 Jan 2;10(1):e1003843. doi: 10.1371/journal.ppat.1003843 (PMC3879355; doi:10.1371/journal.ppat.1003843)
Supplement: Figure S1 — Determination of cross-reactive antibodies to PR8 and P. chabaudi . Graphs in A and B plot the mean O.D. (405 nm) values obtained by ELISA after incubating naïve mouse sera (), hyperimmune anti-P. chabaudi sera (), and anti-PR8 sera (○) with either HA or parasite lysate. Results were obtained from one experiment performed in duplicates. Error bars indicate mean ± S.E. In A, only PR8 sera contained antibodies binding to PR8 HA. In B, P. chabaudi sera and, to a lesser extent, PR8 sera, contained antibodies binding to parasite lysate. C. Three different preparations of bromelain-digested PR8 HA and P. chabaudi lysate were resolved on NuPAGE 12% bis-Tris gels alongside 1× SeeBlue Plus2-prestained standard, electrophorated on Hybond C membrane and probed with normal mouse sera (left panel), hyperimmune P. chabaudi sera (middle panel), and PR8 sera (right panel). No cross-reactive antibodies were detected between P. chabaudi and PR8 sera. The 46 kDa, fragment of HA was only recognised by PR8 sera. D. Graph of the arbitrary virus neutralising Ab titres in naïve mouse sera (), hyperimmune anti-P. chabaudi sera (), and anti-PR8 sera (○). Reactive serum titres of PR8-neutralising antibodies in the serum was measured using a virus neutralising assay. No neutralising antibodies were detected from 1∶2 serial dilutions of 1∶50–1∶51200 in naïve BALB/c serum and anti-P. chabaudi serum. Only PR8 sera were able to neutralise PR8 in vitro. Results were obtained from one experiment performed in duplicates. Error bars indicate mean ± S.E. (PDF) [file ppat.1003843.s001.pdf]

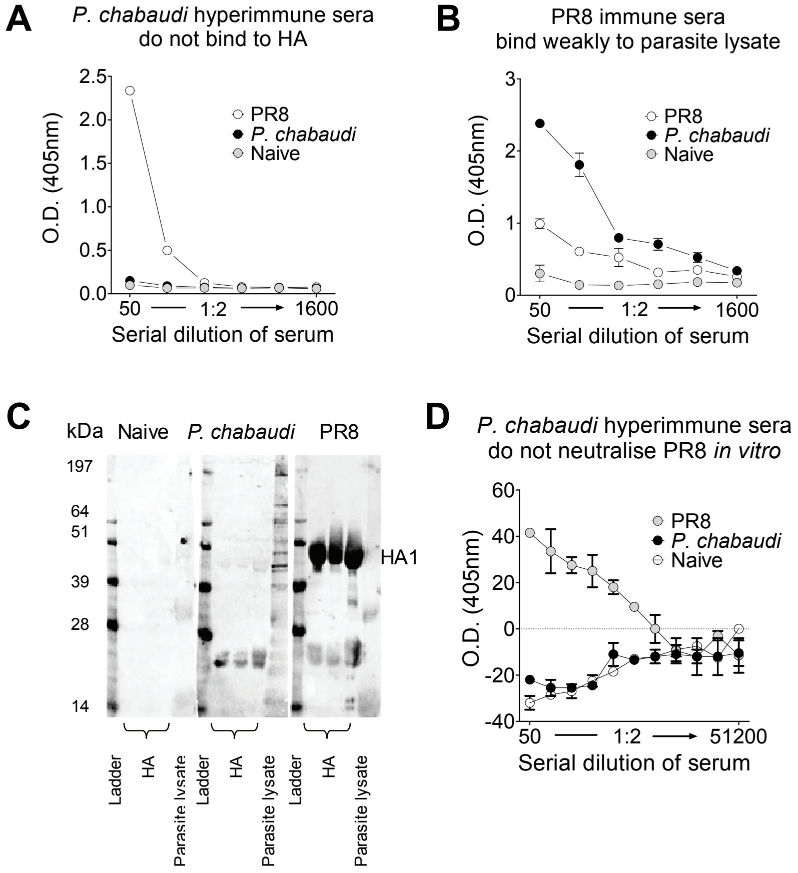

Figure S1. Determination of cross-reactive antibodies to PR8 and *P. chabaudi.*
Graphs in **A** and **B** plot the mean O.D. (405nm) values obtained by ELISA after incubating naïve mouse sera (●), hyperimmune anti-*P. chabaudi* sera (●), and anti-PR8 sera (○) with either HA or parasite lysate. Results were obtained from one experiment performed in duplicates. Error bars indicate mean ± S.E. In **A**, only PR8 sera contained antibodies binding to PR8 HA. In **B**, *P. chabaudi* sera and, to a lesser extent, PR8 sera, contained antibodies binding to parasite lysate. **C.** Three different preparations of bromelain-digested PR8 HA and *P. chabaudi* lysate were resolved on NuPAGE 12% bis-Tris gels alongside 1x SeeBlue Plus2-prestained standard, electrophorated on Hybond C membrane and probed with normal mouse sera (left panel), hyperimmune *P. chabaudi* sera (middle panel), and PR8 sera (right panel). No cross-reactive antibodies were detected between *P. chabaudi* and PR8 sera. The 46 kDa, fragment of HA was only recognised by PR8 sera. **D.** Graph of the arbitrary virus neutralising Ab titres in naïve mouse sera (●), hyperimmune anti-*P. chabaudi* sera (●), and anti-PR8 sera (○). Reactive serum titres of PR8-neutralising antibodies in the serum was measured using a virus neutralising assay. No neutralising antibodies were detected from 1:2 serial dilutions of 1:50 – 1:51200 in naïve BALB/c serum and *anti-P. chabaudi* serum. Only PR8 sera were able to neutralise PR8 *in vitro*. Results were obtained from one experiment performed in duplicates. Error bars indicate mean ± S.E.
