# Supplementary material for: Recovery of an Antiviral Antibody Response following Attrition Caused by Unrelated Infection
Source: PLoS Pathog. 2014 Jan 2;10(1):e1003843. doi: 10.1371/journal.ppat.1003843 (PMC3879355; doi:10.1371/journal.ppat.1003843)
Supplement: Figure S2 — No difference in half-life of serum antibody during acute or chronic infection with P. chabaudi . A. A schematic of the experiment. 8–10 week old naïve female BALB/c mice were infected with P. chabaudi. 24 h or 60 days post-infection, mice were injected i.p. with 200 µg of anti-TNP mIgG2a grown from the Hy1.2 hybridoma. Serum was obtained at various time points after injection. B. Concentration of TNP-specific mIgG2a in serum was quantified by ELISA throughout acute P. chabaudi infection (d1 post-infection) ) or chronic infection (d60 post-infection) () and compared with uninfected age-matched controls (○). Graph showing the mean of data obtained from 2 independent experiments with 5 mice per group. Linear regression was used to find the relationship between the logarithm of serum TNP-specific antibody concentration and time since injection. Antibody half-life was then determined using the equation t1/2 = (ln 2)/κ where κ is the decay constant given by the slope of the best fitting linear function. (PDF) [file ppat.1003843.s002.pdf]

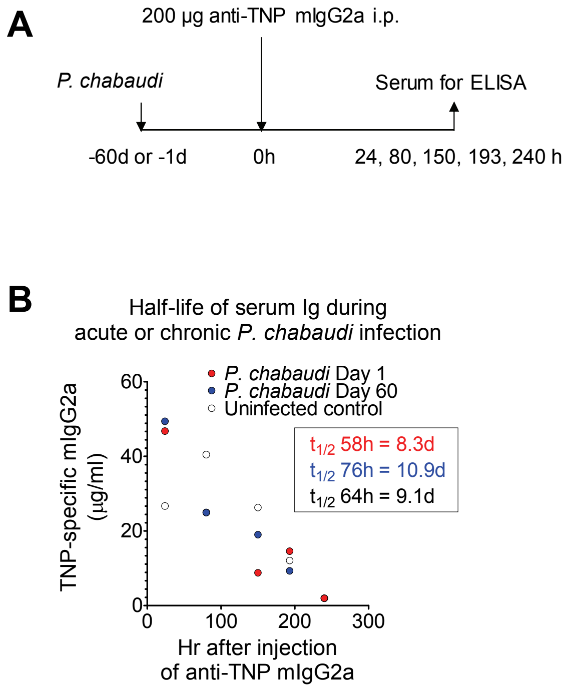

Figure S2. No difference in half-life of serum antibody during acute or chronic infection with *P. chabaudi*.

A. A schematic of the experiment. 8-10 week old naïve female BALB/c mice were infected with *P. chabaudi*. 24h or 60 days post-infection, mice were injected i.p. with 200µg of anti-TNP mIgG2a grown from the Hy1.2 hybridoma. Serum was obtained at various time points after injection. **B.** Concentration of TNP-specific mIgG2a in serum was quantified by ELISA throughout acute *P. chabaudi* infection (d1 post-infection) (●) or chronic infection (d60 post-infection) (●) and compared with uninfected age-matched controls (○). Graph showing the mean of data obtained from 2 independent experiments with 5 mice per group. Linear regression was used to find the relationship between the logarithm of serum TNP-specific antibody concentration and time since injection. Antibody half-life was then determined using the equation $t_{1/2}=(\ln 2)/\kappa$ where κ is the decay constant given by the slope of the best fitting linear function.
