# Supplementary material for: Recovery of an Antiviral Antibody Response following Attrition Caused by Unrelated Infection
Source: PLoS Pathog. 2014 Jan 2;10(1):e1003843. doi: 10.1371/journal.ppat.1003843 (PMC3879355; doi:10.1371/journal.ppat.1003843)
Supplement: Figure S3 — Movement of B220+ CD138+ migratory plasmablasts and B220− CD138+ LLPC in bone marrow during P. chabaudi infection. 8–10 wk old female BALB/c mice were infected by intranasal instillation of 250 HAU of PR8. 150 days later, mice were infected with 105 P. chabaudi pRBCs. Bone marrow was obtained from femur pairs were obtained at various time points after P. chabaudi infection. Migratory plasmablasts (B220+ CD138+) and long-lived plasma cells (B220− CD138+) were quantified using flow cytometry. A. Representative FACs plots showing B220 vs CD138 staining of live gated bone marrow cells in naïve BALB/c mice, 150 days after PR8 infection and 12 days after subsequent P. chabaudi infection. B. Mean and standard error of absolute number per femur pair of migratory plasmablasts (○ B220+ CD138+) and LLPC ( B220− CD138+) on days 0, 8, 10, 12, 25, 45 and 75 after P. chabaudi infection. Data was obtained from one experiment with 3 mice per time point. Error bars indicate median ± error. C. Spleen, PBMC and bone marrow were obtained from femur pairs were obtained on days 0, 7, 8, 10 and 12 after P. chabaudi infection. Representative FACS plots of B220 vs. isotype control and B220 vs. CD138 on spleen, PBMC and bone marrow cells 150 days after PR8 infection and 10 days after subsequent P. chabaudi infection. Relative mean fluorescence indexes of CXCR4, CXCR5, CD19 and MHC class II were determined by multi-parameter flow cytometry on B220+ splenic B cells; B220+ CD138+ splenic plasmablasts; B220+ CD138+ migratory plasmablasts in PMBC; B220+ CD138+ migratory plasmablasts in bone marrow; and B220− CD138+ long-lived plasma cells in bone marrow on day 10 of P. chabaudi infection. D. PBMC were obtained on days 8 and 10 after P. chabaudi infection. HA-specific and total IgG antibody-secreting cells (ASCs) in PBMC in mice infected with PR8 (○) or PR8-P. chabaudi () were quantified using ELISpot. Data was obtained from one experiment with 4 mice per time point. Line indicates the mean value. [file ppat.1003843.s003.pdf]

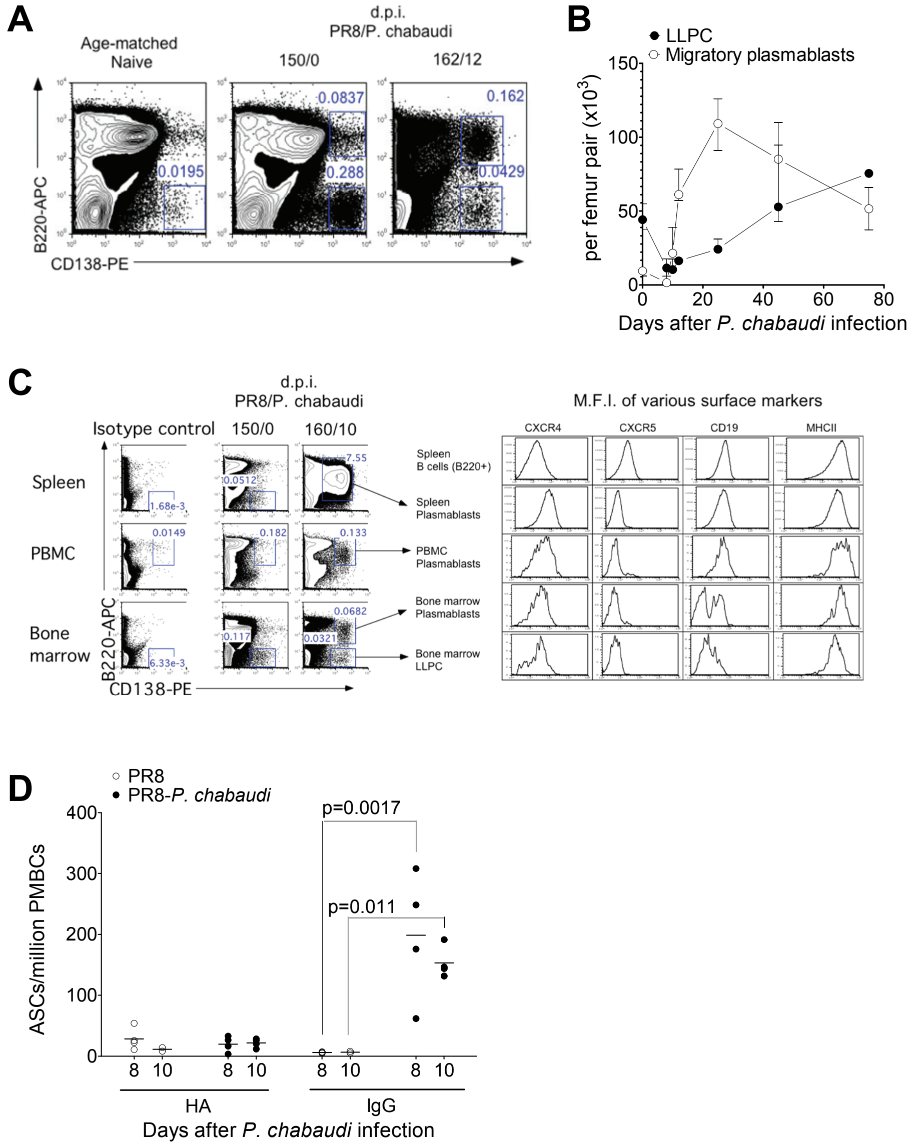

Figure S3. Movement of B220^{+} CD138^{+} migratory plasmablasts and B220^{-} CD138^{+} LLPC in bone marrow during *P. chabaudi* infection.

8-10 wk old female BALB/c mice were infected by intranasal instillation of 250 HAU of PR8. 150 days later, mice were infected with 10^5 *P. chabaudi* pRBCs. Bone marrow was obtained from femur pairs were obtained at various time points after *P. chabaudi* infection. Migratory plasmablasts (B220^{+} CD138^{+}) and long-lived plasma cells (B220^{-} CD138^{+}) were quantified using flow cytometry. **A.** Representative FACs plots showing B220 vs CD138 staining of live gated bone marrow cells in naïve BALB/c mice, 150 days after PR8 infection and 12 days after subsequent *P. chabaudi* infection. **B.** Mean and standard error of absolute number per femur pair of migratory plasmablasts (○ B220^{+} CD138^{+}) and LLPC (● B220^{-} CD138^{+}) on days 0, 8, 10, 12, 25, 45 and 75 after *P. chabaudi* infection. Data was obtained from one experiment with 3 mice per time point.

Error bars indicate median ± error. **C.** Spleen, PBMC and bone marrow were obtained from femur pairs were obtained on days 0, 7, 8, 10 and 12 after *P. chabaudi* infection. Representative FACS plots of B220 vs. isotype control and B220 vs. CD138 on spleen, PBMC and bone marrow cells 150 days after PR8 infection and 10 days after subsequent *P. chabaudi* infection. Relative mean fluorescence indexes of CXCR4, CXCR5, CD19 and MHC class II were determined by multi-parameter flow cytometry on $B220^+$ splenic B cells; $B220^+$ $CD138^+$ splenic plasmablasts; $B220^+$ $CD138^+$ migratory plasmablasts in PMBC; $B220^+$ $CD138^+$ migratory plasmablasts in bone marrow; and $B220^-$ $CD138^+$ long-lived plasma cells in bone marrow on day 10 of *P. chabaudi* infection. **D.** PBMC were obtained on days 8 and 10 after *P. chabaudi* infection. HA-specific and total IgG antibody-secreting cells (ASCs) in PBMC in mice infected with PR8 (○) or PR8-*P. chabaudi* (●) were quantified using ELISpot. Data was obtained from one experiment with 4 mice per time point. Line indicates the mean value. Statistical values were calculated using the Mann-Whitney test.
