# Supplementary material for: Recovery of an Antiviral Antibody Response following Attrition Caused by Unrelated Infection
Source: PLoS Pathog. 2014 Jan 2;10(1):e1003843. doi: 10.1371/journal.ppat.1003843 (PMC3879355; doi:10.1371/journal.ppat.1003843)
Supplement: Figure S4 — Development of hyperimmunoglobulinaemia (IgG) during P. chabaudi infection. 8–10 wk old female BALB/c mice were infected by intranasal instillation of 250 HAU of PR8. 150 days later, mice were infected with 105 P. chabaudi pRPBCs i.p. A. P. chabaudi parasite lysate-specific IgG and B total serum IgG at various time points for up to 60 days after P. chabaudi infection in PR8-P. chabaudi-infected mice () and age-matched control PR8-only mice (○). Each data point represents one mouse from one experiment with 3–5 mice per time point. (PDF) [file ppat.1003843.s004.pdf]

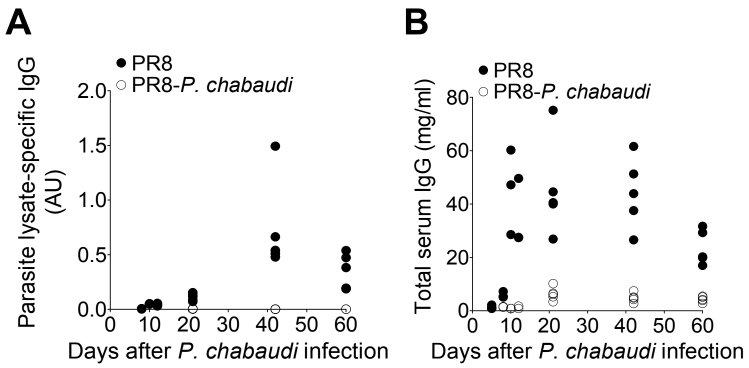

Figure S4. Development of hyperimmunoglobulinaemia (IgG) during *P. chabaudi* infection.
8-10 wk old female BALB/c mice were infected by intranasal instillation of 250 HAU of PR8. 150 days later, mice were infected with 10^5 *P. chabaudi* pRPBCs i.p. **A.** *P. chabaudi* parasite lysate-specific IgG and **B.** total serum IgG at various time points for up to 60 days after *P. chabaudi* infection in PR8-*P. chabaudi*-infected mice (●) and age-matched control PR8-only mice (○). Each data point represents one mouse from one experiment with 3-5 mice per time point.
