# Supplementary material for: Recovery of an Antiviral Antibody Response following Attrition Caused by Unrelated Infection
Source: PLoS Pathog. 2014 Jan 2;10(1):e1003843. doi: 10.1371/journal.ppat.1003843 (PMC3879355; doi:10.1371/journal.ppat.1003843)
Supplement: Figure S5 — Development of HA-specific IgG and parasitaemia in FcγRI,II,III−/− and C57BL/6 mice. 8–10 wk old female FcγRI,II,III−/− and C57BL/6 were infected by intranasal instillation of 250 HAU of PR8. 150 days later, mice were infected with 105 P. chabaudi pRBCs i.p. A. Concentration of HA-specific serum IgG in FcγRI,II,III−/− (○) and C57BL/6 () mice on days 28, 56, 84 and 150 after PR8 infection. Line indicates the median value. B. % parasitaemia throughout acute P. chabaudi infection in FcγRI,II,III−/− (○) and C57BL/6 () mice, when P. chabaudi infection was initiated 150 days after PR8 infection. Graphs indicate the geometric mean ± error of 4 mice per time point. c) % parasitaemia at d8 of P. chabaudi infection in FcγRIIB−/− (○) and C57BL/6 () mice, when P. chabaudi infection was initiated 56 days after PR8 infection. (PDF) [file ppat.1003843.s005.pdf]

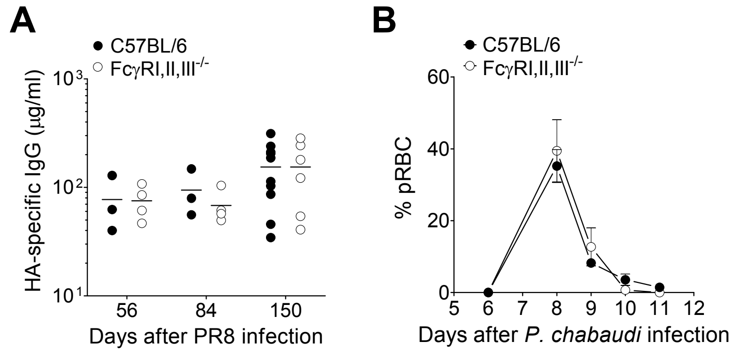

Figure S5. Development of HA-specific IgG and parasitaemia in FcγRI,II,III$^{-/-}$ and C57BL/6 mice. 8-10 wk old female FcγRI,II,III$^{-/-}$ and C57BL/6 were infected by intranasal instillation of 250 HAU of PR8. 150 days later, mice were infected with 10^5 *P. chabaudi* pRBCs i.p. **A.** Concentration of HA-specific serum IgG in FcγRI,II,III$^{-/-}$ (○) and C57BL/6 (●) mice on days 28, 56, 84 and 150 after PR8 infection. Line indicates the median value. **B.** % parasitaemia throughout acute *P. chabaudi* infection in FcγRI,II,III$^{-/-}$ (○) and C57BL/6 (●) mice, when *P. chabaudi* infection was initiated 150 days after PR8 infection. Graphs indicate the geometric mean ± error of 4 mice per time point.
