# Supplementary material for: Recovery of an Antiviral Antibody Response following Attrition Caused by Unrelated Infection
Source: PLoS Pathog. 2014 Jan 2;10(1):e1003843. doi: 10.1371/journal.ppat.1003843 (PMC3879355; doi:10.1371/journal.ppat.1003843)
Supplement: Figure S6 — Depletion efficacy of treatment with 2H7 mAb in hCD20tg/BALB/c mice. 8–10 wk old female hCD20tg and hCD20tg-negative littermates were infected by intranasal instillation of 250 HAU of Influenza A/PR/8/34. Venous blood was obtained at various time points up to 150 days post-infection and processed for serum. A. The concentration of HA-specific IgG in serum from hCD20tg [○; n = 9(d28), 9(d56), 5(d84) and 13(d150)] and hCD20tg-negative littermates [; n = 8(d28), 8(d56), 8(d84) and 13(d150)] were quantified by ELISA. Line indicates median values. B. Schematic representation of experiment. 8–10 wk old female hCD20tg and hCD20tg-negative littermates and BALB/c WT mice were infected by intranasal instillation of 250 HAU of Influenza A/PR/8/34. 150 days post-infection, mice were treated with 0.5 mg/wk of 2H7/saline i.p every 48 hours for 2 weeks (2 mg/wk). Analysis of efficacy of depletion was done 1 day post-depletion. Assessment of persistence of specific plasma cells and serum antibody was done 42, 84 and 112 days post-depletion by ELISA, flow cytometry and ASC and memory B cell ELIspot. C. 1 day post-depletion, spleens were obtained and stained with surface markers as indicated for IgD+, CD21+ and CD19+ B cells. These markers were co-expressed on the majority of B cells. D. Graphs show individual data points of the total number of IgD+, CD21+ and CD19+ B cells in spleens before treatment and after anti-hCD20 mAb treatment in hCD20tg [○; n = 7(pre-treatment), 5(d1); 5(d42); 3(d150)] and hCD20tg-negative [; n = 5(pre-treatment), 2(d1); 3(d42); 3(d150)] mice. E. Graphs show individual data points of the total number of CD138+ B220− LLPCs in spleen and bone marrow before treatment and after anti-hCD20 mAb treatment in hCD20tg [○; n = 7(pre-treatment), 5(d1); 5(d42); 3(d150)] and hCD20tg-negative [; n = 5(pre-treatment), 2(d1); 3(d42); 3(d150)] mice. F. Graph shows individual data points of the total number of B220+ IgD− GL7+ CD38− GC B cells in spleen before treatment and a [file ppat.1003843.s006.pdf]

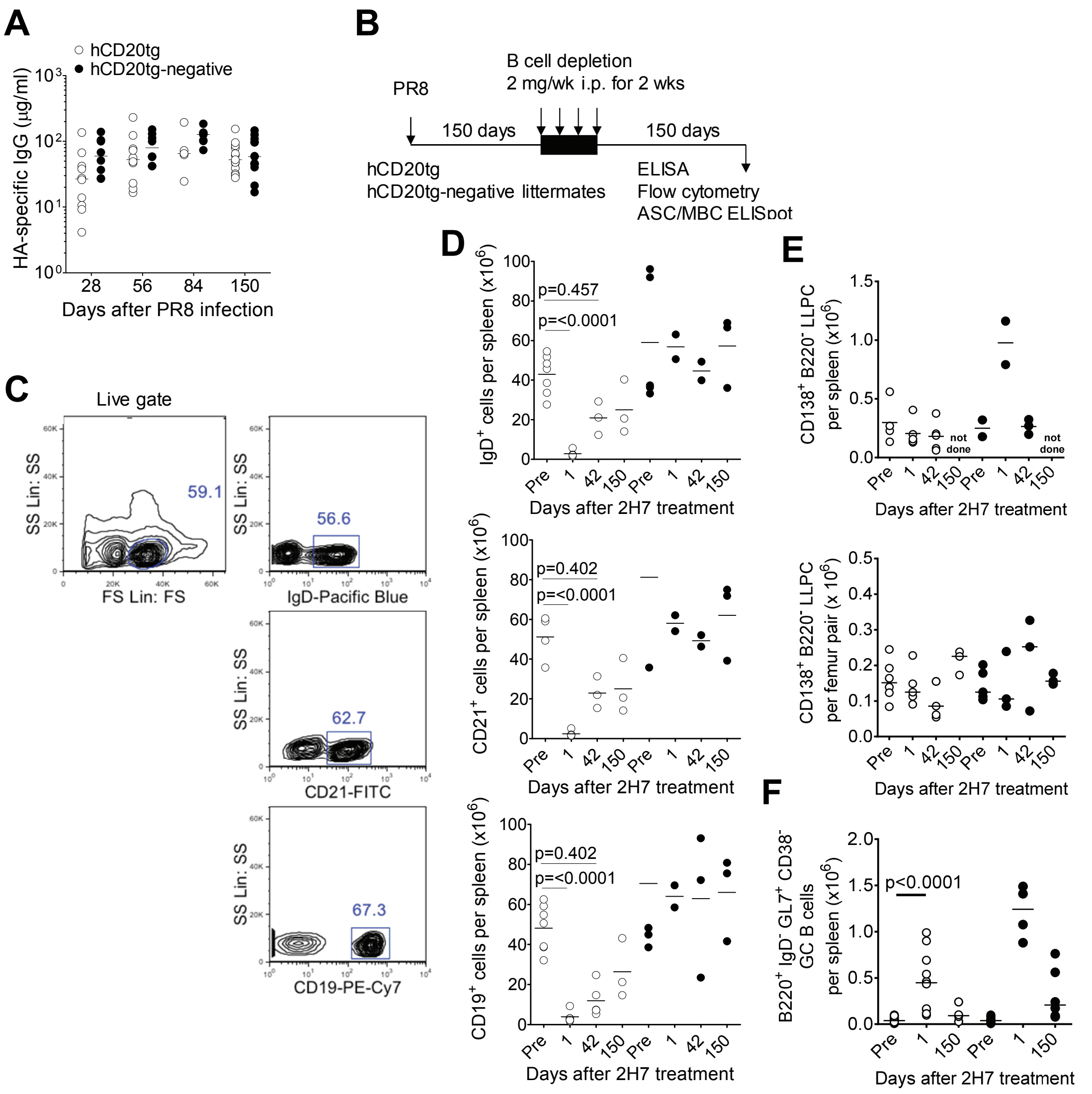

Figure S6. Depletion efficacy of treatment with 2H7 mAb in hCD20tg/BALB/c mice
8-10 wk old female hCD20tg and hCD20tg-negative littermates were infected by intranasal instillation of 250 HAU of Influenza A/PR/8/34. Venous blood was obtained at various time points up to 150 days post-infection and processed for serum. **A.** The concentration of HA-specific IgG in serum from hCD20tg [○; n=9(d28), 9(d56), 5(d84) and 13(d150)] and hCD20tg-negative littermates [●; n=8(d28), 8(d56), 8(d84) and 13(d150)] were quantified by ELISA. Line indicates median values. **B.** Schematic representation of experiment. 8-10 wk old female hCD20tg and hCD20tg-negative littermates and BALB/c WT mice were infected by intranasal instillation of 250 HAU of Influenza A/PR/8/34. 150 days post-infection, mice were treated with 0.5 mg/wk of 2H7/saline i.p every 48 hours for 2 weeks (2mg/wk). Analysis of efficacy of depletion was done 1 day post-depletion. Assessment of persistence of specific plasma cells and serum antibody was done 42, 84 and 112 days post-depletion by ELISA, flow cytometry

and ASC and memory B cell ELIspot. **C.** 1 day post-depletion, spleens were obtained and stained with surface markers as indicated for IgD$^+$, CD21$^+$ and CD19$^+$ B cells. These markers were co-expressed on the majority of B cells. **D.** Graphs show individual data points of the total number of IgD$^+$, CD21$^+$ and CD19$^+$ B cells in spleens before treatment and after anti-hCD20 mAb treatment in hCD20tg [○; n=7(pre-treatment), 5(d1); 5(d42); 3(d150)] and hCD20tg-negative [●; n=5(pre-treatment), 2(d1); 3(d42); 3(d150)] mice. **E.** Graphs show individual data points of the total number of CD138$^+$ B220$^-$ LLPCs in spleen and bone marrow before treatment and after anti-hCD20 mAb treatment in hCD20tg [○; n=7(pre-treatment), 5(d1); 5(d42); 3(d150)] and hCD20tg-negative [●; n=5(pre-treatment), 2(d1); 3(d42); 3(d150)] mice. **f)** Graph shows individual data points of the total number of B220+ IgD- GL7+ CD38- GC B cells in spleen before treatment and after anti-hCD20 mAb treatment in hCD20tg [○; n=8(pre-treatment), 5(d1); 3(d150)] and hCD20tg-negative [●; n=6(pre-treatment), 2(d1); 3(d150)] mice. Line indicates the median value. Statistical values were calculated using the Mann-Whitney test. Statistical values were calculated using the Mann-Whitney test using data pooled from 2 independent experiments. The *n* number of hCD20tg-negative mice was too small for Mann-Whitney test to be performed.
